# The roles of arbuscular mycorrhizal fungi (AMF) in phytoremediation and tree-herb interactions in Pb contaminated soil

**DOI:** 10.1038/srep20469

**Published:** 2016-02-04

**Authors:** Yurong Yang, Yan Liang, Xiaozhen Han, Tsan-Yu Chiu, Amit Ghosh, Hui Chen, Ming Tang

**Affiliations:** 1 State Key Laboratory of Soil Erosion and Dryland Farming on the Loess Plateau, Northwest A&F University, Yangling, Shaanxi 712100, China; 2 College of Forestry, Northwest A&F University, Yangling, Shaanxi 712100, China; 3 Joint BioEnergy Institute, 5885 Hollis St, Emeryville, CA 94608, USA; 4 Physical Biosciences Division, Lawrence Berkeley National Laboratory, Berkeley, CA 94720, USA; 5 Plant Systems Biology Lab, Botany and Plant Science, School of Natural Sciences, National University of Ireland, Galway, Ireland; 6 School of Energy Science and Engineering, PK Sinha Centre for Bioenergy, Indian Institute of Technology Kharagpur, Kharagpur 721302, India

## Abstract

Understanding the roles of arbuscular mycorrhizal fungi (AMF) in plant interaction is essential for optimizing plant distribution to restore degraded ecosystems. This study investigated the effects of AMF and the presence of legume or grass herbs on phytoremediation with a legume tree, *Robinia pseudoacacia*, in Pb polluted soil. In monoculture, mycorrhizal dependency of legumes was higher than that of grass, and AMF benefited the plant biomass of legumes but had no effect on grass. Mycorrhizal colonization of plant was enhanced by legume neighbors but inhibited by grass neighbor in co-culture system. N, P, S and Mg concentrations of mycorrhizal legumes were larger than these of non-mycorrhizal legumes. Legume herbs decreased soil pH and thereby increased the Pb concentrations of plants. The neighbor effects of legumes shifted from negative to positive with increasing Pb stress levels, whereas grass provided a negative effect on the growth of legume tree. AMF enhanced the competition but equalized growth of legume-legume under unpolluted and Pb stress conditions, respectively. In conclusion, (1) AMF mediate plant interaction through directly influencing plant biomass, and/or indirectly influencing plant photosynthesis, macronutrient acquisition, (2) legume tree inoculated with AMF and co-planted with legume herbs provides an effective way for Pb phytoremediation.

The biosphere pollution with toxic metals due to anthropogenic activities poses a serious environmental and human health problem. As one of the most abundant, ubiquitously distributed toxic elements in soil, lead (Pb) exerts adverse effect on both terrestrial and aquatic ecosystems^1^. In 2014, the production of recoverable Pb from mining operations reached to 2950, 720, and 355 thousand metric tons in China, Australia, and USA, respectively (USGS 2014). High level of Pb can cause serious harm to plant growth and development, photosynthesis, water balance, and nutrient capture^1^. However, some plant species can still survive, grow and reproduce in environments polluted with heavy metals (HMs), indicating that they developed certain mechanisms to combat with such adverse environmental conditions. Phytoremediation, the technique of using the plant species, named hyperaccumulators, which are capable of accumulating huge amount of HM in their tissues without obvious toxicity symptoms to cleanup HM pollutants, provides a promising future in ecological restoration of mine area and remediation of polluted soil^2^. However, the application of hyperaccumulators is limited by their slow growth rate, small biomass and long period of time as required for cleaning up HMs in soil.

There are many ways to overcome the limitations of phytoremediation with hyperaccumulators. One of them is the use of other plant species with lower capacity of HM accumulation but fast-growth rate and high biomass. The high biomass of plants can compensate for the relatively low capacity for HM accumulation. Another strategy to increase the efficiency of phytoremediation is to inoculate phytoremediation plants with arbuscular mycorrhizal fungi (AMF)^3^. AMF are one of the most important soil microorganisms that develop mutual symbiotic association with roots of most terrestrial plants^4^. They may facilitate host plants to uptake and transport phosphorus (P) and other relatively immobile soil nutrients, promote plants growth and enhance their stress tolerance^4^.

In the current research, we studied the efficiency and mechanism of phytoremediation with woody legume *Robinia pseudoacacia* L. inoculated with AMF. *R. pseudoacacia* have been suggested to have the potential for extracting metal contaminants from soil due to its fast growth, high biomass, capacity of accumulating large amounts of HM, and atmospheric nitrogen (N) fixation^5^. The AMF species, *Rhizophagus intraradices* (N.C. Schenck & G.S. Sm.) C. Walker & A. Schüßler (formerly known as *Glomus intraradices*), was selected for legume inoculation based on our previous investigation suggesting its dominant presence in HM contaminated soil^6^. The fungus can colonize a broad range of plant species, including grass, clover, legume tree, etc.^7^,^8^. In addition, Red clover (*Trifolium pratense* L.), Alfalfa (*Medicago sativa* L.) and Ryegrass (*Lolium perenne* L.) were planted as neighboring herbs to the legume tree *R. pseudoacacia*. The three herbal plant species were known to be the most important pasture and ground cover herbs distributed all over the world in various ecosystems^9^. Co-planting with neighboring plants was included in the study because plant interactions exist universally in natural and cultivated ecosystem. Interactions between neighboring plants are recognized as one of the most important factors determining dynamics of plant populations, the structure of plant communities, as well as their evolutions^10^. Plant-plant interactions include competition and facilitation, or, the co-existence of the two relationships. The sign and strength of plant-plant interactions can be altered by both biotic and abiotic context^11^. The stress-gradient hypothesis (SGH) stated that plant-plant competition and facilitation could be shifted by environmental conditions. Competition usually dominates in benign environments and plays an important role in changing plant diversity and community, whereas facilitation may contribute more than competition to plant distribution in severe environments^12^.

Although the relationship between plant interactions and abiotic stress gradients has been explored in a wide range of gradients^12^, the effects of AMF and Pb pollution on plant interaction have not been well studied. The extensive AM hyphal networks underground provide a direct physical link between soils and the roots of multiple host plants, which potentially mediates plant-plant interactions through transferring nutrients, carbon and water from one plant to another^4^. Therefore, AMF have been shown to probably play an important role in the formation and maintenance of plant distribution and community composition^13^,^14^. Nevertheless, excessive Pb not only strongly inhibit the growth and development of plants but also decrease the diversity and activity of soil microbial community, resulting in low AMF growth rate, colonization rate and spore density^5^. Thus, the diversity, composition structure and process of the plant community might be changed by Pb pollution through affecting AMF hyphal networks and the interaction between host plant and AMF. Qiao *et al*.^15^ indicated that the effects of AMF on competition between faba bean and wheat were partly attributed to enhancement of temporal N and P acquisition. However, the uptake of nutrients might vary in different plants and HM can exhibit influences on the relationship between AMF and host plant. Furthermore, the tolerance to HM varies significantly among plant species even under the same stress condition, which would modify the SGH under Pb stress gradient to a certain extent. When different plants are grown together, AMF growth and sporulation will become host preference^16^, resulting in various mycorrhizal dependencies (MDs) and changes of plant-plant interactions.

In summary, the current study set up a model system, with different legume tree-herbs-AMF-Pb combinations, to study the effects of AMF and Pb on phytoremediation and the dynamic interactions between tree and herbs, which has not been well documented before. Furthermore, to use phytoremediation technology in a three-dimensional scale, it is very important to understand the tree-herb interactions: the HMs in the shallow soil layer are extracted and/or immobilized by herbaceous plants with rapid growth and high biomass, while the HMs flowing to the deep soil layer are extracted, filtrated and/or stabilized by tree species with deep root systems to prevent groundwater from pollution (Fig. 1). Therefore, the specific objectives were to (1) evaluate the effects of Pb stress and AMF inoculation on the growth, Pb accumulation, macronutrient concentrations of herbs (*T. pratense, M. sativa, L. perenne*) and legume tree (*R. pseudoacacia*), (2) investigate whether and how the Pb stress, AM inoculation and different herbs regulate the Pb accumulative characteristics of *R. pseudoacacia* and the interactions among neighboring plants, (3) evaluate whether the influence of AMF tree-herb interactions in Pb contaminated soil is consistent with SGH or provides further extension of the SGH.

## Results

### Mycorrhizal colonization (MC)

**Figure 2:**
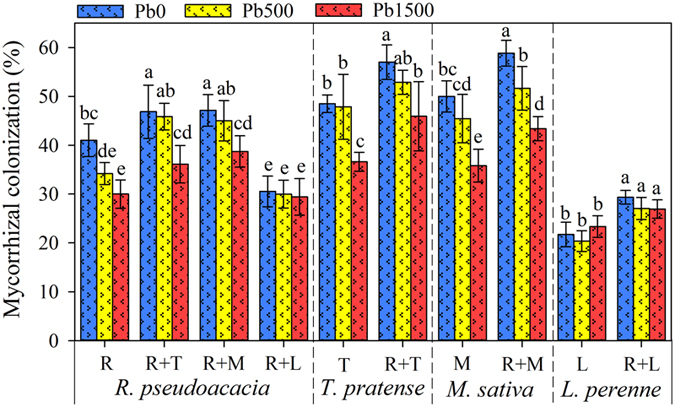
The mycorrhizal colonization (MC) of *R. pseudoacacia, T. pretense, M. sativa* and *L. perenne* was affected by Pb stress levels. Pb0, Pb500 and Pb1500 represent Pb concentration of 0, 500 and 1500 mg kg^−1^, respectively. Monocultures of *R. pseudoacacia, T. pretense, M. sativa* and *L. perenne* are represented by R, T, M and L, respectively. The co-culture planting pattern is represented by the two co-culture species connected with the plus sign (+). The results are reported as the mean (n = 4) ± SD. Different letters indicate that significant differences were detected in the MC of each plant species grown in one planting pattern but at different Pb levels by Duncan’s multiple-range tests (*P* < 0.05).

Microscopic assessment confirmed that efficient symbiosis was established between AMF and roots of plants. Under control treatments, almost no MC could be detected in non-inoculated plants (<1%). The residue level of MC in non-mycorrhizal plants (<1%) was likely to be false positive (a small amount of stain was randomly retained by the root hairs, vessel cells. etc.) when scoring root slides. When grown in monoculture patterns, legumes (*R. pseudoacacia, T. pretense* and *M. sativa*) had much higher MC compared to grass species (*L. perenne*) in all treatments, whereas no difference was found in MC between *T. pretense* and *M. sativa* at all Pb stress levels (Fig. 2; Tables S2, S3). The highest Pb stress level (Pb1500, 1500 mg kg^−1^ Pb) greatly decreased the MC of legumes; however, there was no effect of Pb addition on MC of *L. perenne* in all treatments (Fig. 2; Tables S2, S3). It is interesting to note that plants had different effects on MC of neighboring plants in co-culture patterns. The presences of legumes (*R. pseudoacacia, T. pretense* or *M. sativa*) significantly increased MC of co-cultured plants (*R. pseudoacacia* or *L. perenne*), while the presence of grass (*L. perenne*) significantly reduced the MC of *R. pseudoacacia* (*F* = 16.43, *P* = 0.00) (Fig. 2; Tables S2, S3). The maximum MC was observed in the roots of *M. sativa* (58.84%, in “R + M” planting pattern) followed by *T. pretense* (57.02%, in “R + T” planting pattern) at Pb0 (0 mg kg^−1^ Pb) level, while the minimum MC was found in the roots of *L. perenne* (20.37%) grown individually at Pb500 (500 mg kg^−1^ Pb) stress level (Fig. 2).

### Total biomass of plants

**Figure 3:**
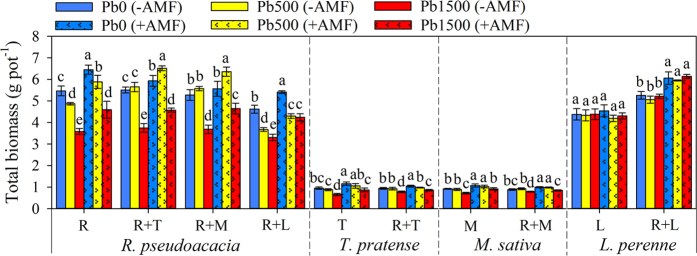
Total biomass of each plant species per pot was affected by Pb stress levels and AMF inoculation. Pb0, Pb500 and Pb1500 represent Pb concentration of 0, 500 and 1500 mg kg^−1^, respectively. Monocultures of *R. pseudoacacia, T. pretense, M. sativa* and *L. perenne* are represented by R, T, M and L, respectively. The co-culture planting pattern is represented by the two co-culture species connected with the plus sign (+). The results are reported as the mean (n = 4) ± SD. Different letters indicate that significant differences were detected in the total biomass of each plant species per pot grown in one planting pattern but at different Pb levels and AMF inoculation status by Duncan’s multiple-range tests (*P* < 0.05).

Total biomass of plants per pot was significantly affected by neighbor presence, AMF inoculation, and Pb level (Fig. 3; Tables S1–S3). The total biomass of legumes was much lower at the highest Pb level (Pb1500) compared to that at the lowest Pb level (Pb0) in all planting patterns, whereas no effects of Pb addition could be found on the total biomass of *L. perenne* grown individually. Non-mycorrhizal legumes were more sensitive to Pb addition than that of mycorrhizal legumes. For example, at Pb1500 stress level, the total biomass reductions of non-mycorrhizal *R. pseudoacacia* were −34.43%, −32.12%, −30.17% and −28.20% for “R”, “R + T”, “R + M”, “R + L” planting patterns, respectively, while total biomass reductions of mycorrhizal *R. pseudoacacia* were −28.84%, −22.93%, −16.37% and −21.63% for “R”, “R + T”, “R + M”, “R + L” planting patterns, respectively.

The presence of AMF greatly increased the total biomass of legumes in all treatments, whereas no effects could be found on the total biomass of *L. perenne* grown individually (Fig. 3; Tables S1–S3). Mycorrhizal *T. pretense, M. sativa* and *L. perenne* had much lower root/shoot ratio compared to non-mycorrhizal plants in “R + T”, “R + M” and “R + L” planting patterns (Table S1). However, AMF presence did not affect the root/shoot ratios of plants grown individually at all Pb levels.

The effects of neighbor presence on total biomass of co-cultured plant highly depended on plant species and Pb stress level (Fig. 3; Tables S2–S3). The total biomass of *R. pseudoacacia* was significantly increased by the presence of *T. pretense* (*F* = 8.50, *P* = 0.01), but greatly reduced by the presence of *L. perenne*. Moreover, the total biomass of *L. perenne* was much higher in “R + L” planting pattern than that in “L” planting pattern. It is interesting to note that the presence of *R. pseudoacacia* significantly decreased the root/shoot ratio of legume herbs (*T. pretense* and *M. sativa*), but had no effect on that of grass (*L. perenne*).

According to the results from multiple ANOVA comparisons, the interactions between neighbor presence and Pb stress level (neighbor × Pb) significantly affected the total biomass of all plants except for *L. perenne* grown individually (Tables S2, S3). However, the interactions among neighbor presence, AMF inoculation and Pb stress level (neighbor × AMF × Pb) had no effect on the total biomass of plants in all planting pattern.

### Macronutrient concentrations

**Figure 4:**
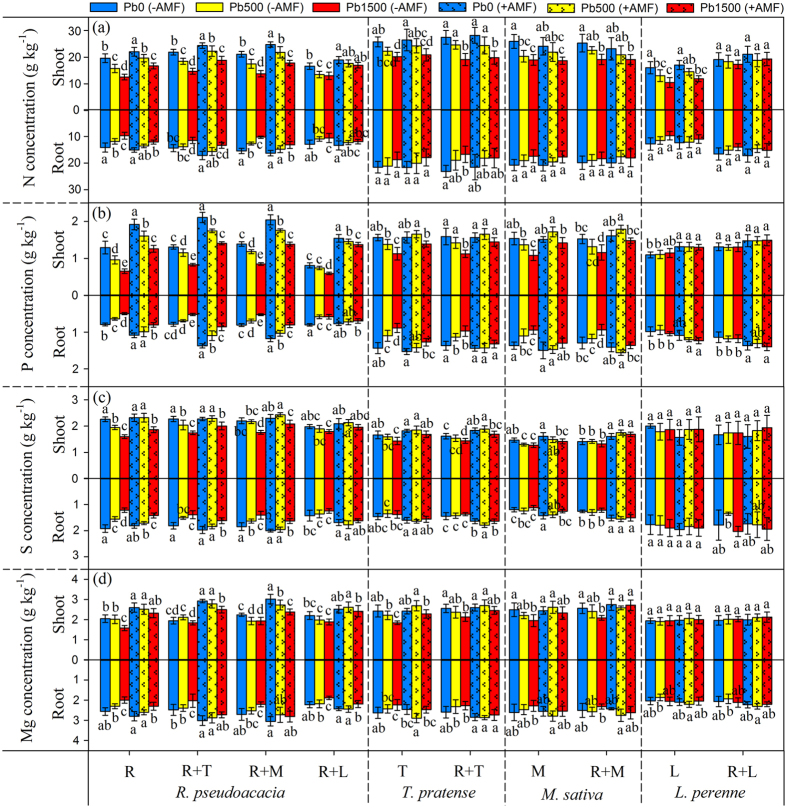
N (nitrogen) (**a**) P (phosphorus) (**b**) S (sulphur) (**c**) and Mg (magnesium) (**d**) concentrations in shoots and roots of *R. pseudoacacia, T. pretense, M. sativa* and *L. perenne* were affected by Pb stress levels and AMF inoculation. Pb0, Pb500 and Pb1500 represent Pb concentration of 0, 500 and 1500 mg kg^−1^, respectively. Monocultures of *R. pseudoacacia, T. pretense, M. sativa* and *L. perenne* are represented by R, T, M and L, respectively. The co-culture planting pattern is represented by the two co-culture species connected with the plus sign (+). The results are reported as the mean (n = 4) ± SD. Different letters indicate that significant differences were detected in the macronutrient concentration of each plant species grown in one planting pattern but at different Pb levels and AMF inoculation status by Duncan’s multiple-range tests (*P* < 0.05).

The macronutrient concentrations in shoots and roots of plants were showed in Fig. 4 and Tables S4–S5, and they were significantly affected by neighbor presence, AMF inoculation, Pb stress level and their interactions (Tables S6–S8). The Pb addition considerably affected the N, P, sulfur (S) and magnesium (Mg) concentrations in both shoots and roots of *R. pseudoacacia* in all planting patterns except for “R + L”. Moreover, the concentrations of N, P and S in shoots of *T. pretense* and *M. sativa* were greatly decreased by Pb addition in all planting patterns, but shoot S concentration of *M. sativa* in “R + M” planting pattern was not affected. However, only shoot N concentration of *L. perenne* was affected by Pb stress levels (Table S8). No effects of Pb addition could be observed on sodium (Na), potassium (K) and calcium (Ca) concentrations in either shoots or roots of *R. pseudoacacia* in all planting patterns (Tables S6, S7).

The presence of AMF increased the N, P, S and Mg concentrations in shoots and roots of *R. pseudoacacia* in all planting patterns, but did not influence its root N concentration in “R + L” planting pattern. The N and P concentrations in both shoots and roots of *T. pretense* and *M. sativa* were greatly increased by AMF inoculation in all treatments, while only P concentration of *L. perenne* was influenced by AMF presence in monoculture planting pattern (Table S8). Furthermore, there were no differences in Na, K, and Ca concentrations between non-mycorrhizal and mycorrhizal *R. pseudoacacia* in all planting patterns (Tables S6, S7).

The effects of neighbor plants on macronutrient concentrations in the shoots and roots of co-cultured plants highly depended on plant species, Pb stress level and their interactions (Tables S6–S8). Legume plants (*T. pretense* and *M. sativa*) showed positive or no effects on N, P, S and Mg concentrations in both shoots and roots of co-cultured plants in all treatments. The grass neighbor (*L. perenne*) firstly presented significantly negative effects on N, P, S and Mg concentrations of *R. pseudoacacia* at Pb0 level, however, with the increase of Pb stress level, the grass neighbor showed slightly positive effects on these nutrient uptakes at the most toxic Pb stress level (Pb1500) (Table S4). In contrast, the presence of *R. pseudoacacia* could significantly increase the N and P concentrations in both shoots and roots of *L. perenne* (Table S8).

The results from multiple ANOVA comparisons analysis showed that only shoot P concentration of *R. pseudoacacia* was significantly influenced by interactions among neighbor presence, AMF inoculation and Pb stress level (neighbor × AMF × Pb). In contrast, the interaction of neighbor × Pb had significant effects on P and S concentrations in both shoots and roots of *R. pseudoacacia*, while neighbor × AMF interaction greatly influenced the shoot N, P and S concentrations of *R. pseudoacacia* (Tables S6, S7). However, no macronutrient concentrations in either shoots or roots of herbs (*T. pretense, M. sativa* or *L. perenne*) could be influenced by the interactions of neighbor × AMF, neighbor × Pb and neighbor × AMF × Pb (Table S8).

### Pb concentration and distribution

**Figure 5:**
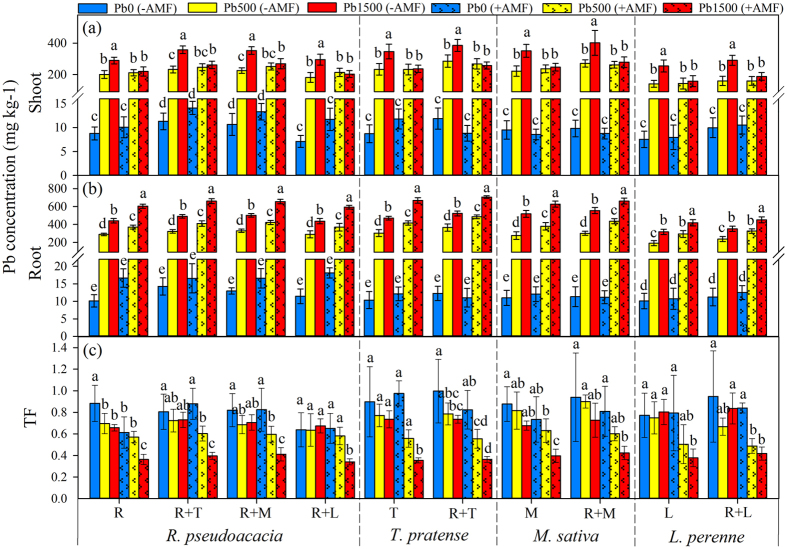
Pb concentrations and translocation factors (TFs) in *R. pseudoacacia, T. pretense, M. sativa* and *L. perenne* were affected by Pb stress levels and AMF inoculation. Pb0, Pb500 and Pb1500 represent Pb concentration of 0, 500 and 1500 mg kg^−1^, respectively. Monocultures of *R. pseudoacacia, T. pretense, M. sativa* and *L. perenne* are represented by R, T, M and L, respectively. The co-culture planting pattern is represented by the two co-culture species connected with the plus sign (+). The results are reported as the mean (n = 4) ± SD. Different letters indicate that significant differences were detected in the Pb concentration or TF of each plant species grown in one planting pattern but at different Pb levels and AMF inoculation status by Duncan’s multiple-range tests (*P* < 0.05).

The Pb concentrations in both shoots and roots of legumes (*R. pseudoacacia, T. pretense* and *M. sativa*) and grass (*L. perenne*) were showed in Fig. 5 and Table S9. Pb accumulation was greater in roots than that in shoots of all plants. The concentration of Pb increased with increasing Pb stress levels. Root Pb concentrations were higher in mycorrhizal plants compared to non-mycorrhizal plants at 500 mg kg^−1^ and 1500 mg kg^−1^ stress levels. In contrast, shoot Pb concentrations in mycorrhizal plants were lower than non-mycorrhizal plants at the highest Pb stress level (Pb1500), whereas, no difference could be found in shoot Pb concentrations between non-mycorrhizal and mycorrhizal plants at 500 mg kg^−1^ Pb stress level (Fig. 5; Table S9). The presence of legume neighbors considerably increased the shoot and root Pb concentrations in both non-mycorrhizal and mycorrhizal plants, whereas, no effects of grass neighbor could be detected on Pb accumulation at all Pb stress levels.

The translocation factors (TFs) of Pb in mycorrhizal plants were significantly lower at 1500 mg kg^−1^ Pb stress level than that at control level in all planting patterns. On the contrary, the TFs in non-mycorrhizal plants did not show any difference along the gradients of Pb in all planting patterns except for the TF of Pb in *R. pseudoacacia* grown individually (Fig. 5; Table S9).

According to the results from multiple ANOVA comparisons (Tables S10, S11), both AMF inoculation and Pb addition had significant effects on Pb concentration and TFs in plants. However, the interactions of neighbor × AMF and neighbor × AMF × Pb did not show any effects on Pb concentration and TFs in all plants no matter in monoculture or co-culture patterns. It is interesting to note that the presence of legume neighbor significantly increased the Pb accumulation in both shoots and roots of plants, but had no effect on TF values at all Pb stress levels (Tables S10, S11).

### The relative interaction index (RII)

**Figure 6:**
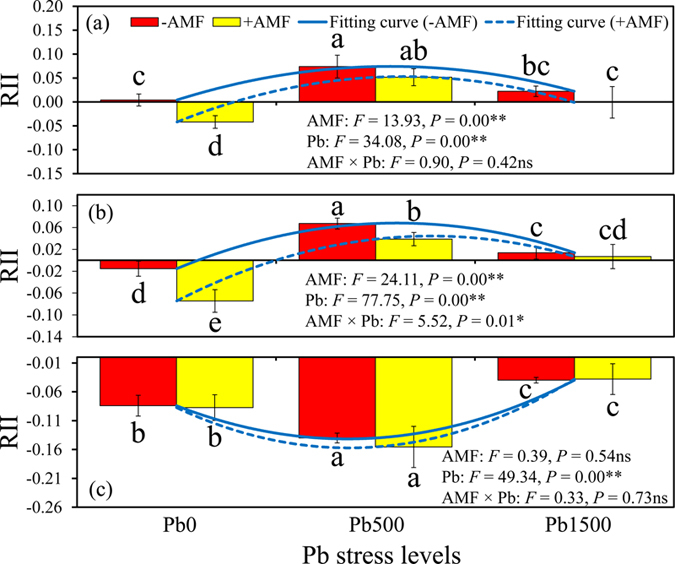
The RII of *T. pretense, M. sativa* and *L. perenne* to legume tree (*R. pseudoacacia*) in “R + T”. (**a**) “R + M” (**b**) and “R + L” (**c**) planting patterns was affected by Pb stress levels and AMF inoculation. The blue solid line (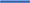) represents the relationship between the RII of non-mycorrhizal plants and the Pb stress gradient; the blue dashed line (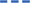) presents the relationship between the RII of mycorrhizal plants and the Pb stress gradient. Pb0, Pb500 and Pb1500 represent Pb concentration of 0, 500 and 1500 mg kg^−1^, respectively. The results are reported as the mean (n = 4) ± SD. Different letters indicate that significant differences were detected in the RII of neighbors to legume tree (*R. pseudoacacia*) in one planting pattern but at different Pb levels and AMF inoculation status by Duncan’s multiple-range tests (*P* < 0.05). Two-way ANOVAs were used to determine the significance of the effects of AMF inoculation (AMF), Pb level (Pb), and their interactions (AMF × Pb) on RII (**P < 0.01; *P < 0.05; ns, no significance).

The neighbor effects as indicated by RII were presented in Fig. 6. The neighbor effects of legume herbs shifted from neutral or negative to positive with increasing Pb stress level, while the presence of grass provided a negative feedback to legume tree at all Pb levels. According to a two-way ANOVA, the RII was significantly affected by AMF inoculation, Pb level, but was not influenced by their interactions (AMF × Pb) except for “R + M” planting pattern. In “R + T” planting pattern, the RII was significantly influenced by AMF inoculation and Pb stress level (*F* = 13.93, *P* = 0.00 for AMF inoculation; *F* = 34.08, *P* = 0.00 for Pb stress level; *F* = 0.90, *P* = 0.42 for AMF × Pb). In “R + M” planting pattern, the RII was also greatly affected by AMF inoculation and Pb level (*F* = 24.11, *P* = 0.00 for AMF inoculation; *F* = 77.75, *P* = 0.00 for Pb stress level; *F* = 5.52, *P* = 0.01 for AMF × Pb). In contrast, the RII was only significantly influenced by Pb stress level (*F* = 49.34, *P* = 0.00) but not AMF inoculation (*F* = 0.39, *P* = 0.54) and their interactions (*F* = 0.33, *P* = 0.73) in “R + L” planting pattern. The effects of neighbor and AMF presences on RII mainly depended on plant species and Pb stress level (Fig. 6). For example, AMF presence significantly reduced RII in “R + T” and “R + M” planting patterns, but had no effect on the RII in “R + L” planting pattern at control Pb level (Pb0). However, with the increase of Pb stress level (Pb500), the RII in “R + T” and “R + M” planting patterns significantly increased, while the RII in “R + L” planting pattern greatly reduced.

## Discussion

AMF compose an important functional group of soil microorganisms in natural ecosystems, by forming symbiotic associations with most terrestrial vascular flowering plants^4^. The underground hyphal networks formed by AMF can influence plant growth, nutrient acquisition as well as plant-plant interactions. However, plant species differ in their dependency on AMF^14^, as reflected by various MC and growth performance of host plants after mycorrhizal inoculation. The MC of legume plants (*R. pseudoacacia, T. pratense* and *M. sativa*) was much higher than that of grass (*L. perenne*) and legume plants were more AMF-dependent compared to grass, which are consistent with previous studies^14^. A possible reason for this phenomenon is that rhizobia share a common cellular program with AMF in the establishment of symbiosis and thus facilitate MC in the roots of legume plants^17^. Furthermore, the presence of legume plants with a large amount of developed roots and high MC is able to accelerate the growth and branching of AMF hyphae^18^, which subsequently increases the MC of neighboring plants. However, we found that the presence of grass showed negative effects on the MC of *R. pseudoacacia* under all Pb stress levels. Generally, grass is a strong competitor in co-culture planting pattern and has the competitive advantage over seedlings of legume plants not only in nutrient acquisition^15^ but also in growth rate and biomass accumulation, leading to a reduction in the leaf net photosynthetic rate (*P*_n_) of legume plants. Bago *et al*.^19^ suggested that between 4% and 20% of the plant’s total photosynthetic products were provided to AMF, which is a fundamental aspect of symbiosis. Thus, the reduction of the leaf photosynthetic rate of *R. pseudoacacia* may in turn decreases the MC due to the reduction of nutrition supply to AMF.

Mycorrhizal associations could greatly enhance host plant HM tolerance by improving plant nutrient acquisition and by influencing the fate of the metals in both the plants and soil^20^. However, the precise role of AMF in Pb accumulation and distribution in plants is still poorly documented. We found that mycorrhizal plants restricted a large amount of Pb in the roots to protect the above-ground parts from damage, which is consistent with the previous study conducted by Diaz *et al*.^21^ who reported that more Pb and Zn were accumulated in the roots of mycorrhizal *Lygeum spartum* and *Anthyllis cytisoides* than non-mycorrhizal plants at different HM stress levels. Göhre and Paszkowski^22^ hypothesized that the fungal vesicle structures of AMF were similar to plant and fungal vacuoles, which are involved in storing toxic compounds, and thereby, provided an additional detoxification mechanism for host plants. Supportively, an increase in the number of fungal vesicles with the increase of Pb stress levels was found in our study (Fig. S2), which might contribute to the large amount of Pb retention in plant roots. Upon transmission electron microscope analysis, our previous study further confirmed the localization of Pb in AMF, including hyphal cell walls, hyphal membranes, vesicular and vacuole membranes, etc.^23^. In addition, the presence of AMF increased the expression of the phytochelatin synthase gene (*PCS1*) in legume plant (*Sophora viciifolia*) exposed to Pb stress^24^, suggesting another mechanism of Pb immobilization in the roots of plants enhanced by mycorrhizal inoculation.

It is interesting to note that legume herbs (*T. pratense* and *M. sativa*) and grass (*L. perenne*) differ in their effects on Pb accumulation and allocation in the shoots and roots of the target plant (*R. pseudoacacia*), indicating the importance of selecting the appropriate neighboring plants to enhance phytoremediation efficiency in Pb contaminated soil. The Pb concentration in tissues especially roots of plants grown in “R + T” and “R + M” planting patterns was much higher compared to these grown individually, whereas the presence of grass (*L. perenne*) did not influence the Pb concentration of co-cultured *R. pseudoacacia*. We further showed that the soil pH was reduced by the presence of legume neighbors in “R + T” and “R + M” planting patterns (Fig. S3). Generally, the active uptake of cations by the plasmalemma of roots involves H^+^  excretion while anion uptake involves OH^−^ or HCO_3_^−^ excretion^25^. Legume plants commonly form symbiotic associations with rhizobia and accumulate most of their N through symbiotic nitrogen fixation. During this process, legume plants take up more cations than anions and release more H^+^  ions from roots to soil, leading to low pH values in both the rhizosphere and bulk soil^26^. As one of the most important environmental factors, soil pH plays an essential role in HM mobility and availability^26^, and low soil pH could greatly enhance the Pb concentrations in both the shoots and roots of plants (Fig. S4). Therefore, if the legume tree (*R. pseudoacacia*) is used for phytoremediation of Pb-contaminated soil, a higher efficiency of Pb removal can be achieved by intercropping it with legume herbs. However, when applying legume herbs as neighboring plants in phytoremediation, caution is needed in selecting proper legume neighbors because the acid production by N_2_-fixing legume plants is variable and dependent on the plant species, nutrient conditions and soil properties^27^.

Both HM pollution and mycorrhizal inoculation are known to affect acquisition and allocation of macronutrient in plants^28^. Excess soil Pb blocks ion absorption at the cell membrane and competes for ion binding sites on the cell wall^29^,^30^, which exerts an adverse influence on plant nutrient uptake from soil. However, this study showed higher N, P and Mg concentrations in mycorrhizal compared to non-mycorrhizal *R. pseudoacacia* in all Pb stress levels, while mycorrhizal inoculation significantly increased the S concentrations of *R. pseudoacacia* only at 500 mg kg^−1^ and 1500 mg kg^−1^ Pb stress levels. Many factors may contribute to the above results. Generally, non-mycorrhizal plants take up P only by the direct pathway via Pi transporters in the epidermis, whereas mycorrhizal plants can acquire P via root epidermal cells and P transporters in hyphae of AMF over longer distance of more than 10 cm from the root surface^31^. Our previous study also indicated that the enhanced host P concentration partly resulted from high fungal alkaline phosphatase activities^32^. Subramanian and Charest^33^ suggested that the hyphae of AMF were able to utilize inorganic N efficiently and transferred more N from soil to plant roots. The ^35^S-labeling experiments indicated that fungal transcriptional regulation played an important role in S transfer to host plants^34^. In contrast to their positive effects on N, P, S and Mg accumulation, mycorrhizal inoculation showed no significant effect on Na, K and Ca concentrations, suggesting that the enhancements of Na, K and Ca in mycorrhizal plants probably depend on plant species, AMF species and soil conditions^35^.

The presence of legume herbs showed beneficial effects on the N and P uptake of target plant (*R. pseudoacacia*). The exudates such as protons, organic anions, amino acids, and enzymes released from the roots of legume plants probably improve soil nutrient availability^36^. In addition, legume plants might facilitate the acquisition of mineral elements from soil to neighboring plants through root-root interactions or root-AMF-root interactions^37^. Andrade *et al*.^37^ reported that the P/Pb ratio in soybean plants had a decreasing trend with the increase of Pb stress level in soil, and mycorrhizal plants had a much higher P/Pb ratio than non-mycorrhizal ones. The beneficial effects of legume neighbors and AMF on N and P uptake can not only improve plant development but also enhance plant Pb resistance, because the excess P easily forms meta-stable compounds with Pb^38^ and thereby significantly reduce the bioavailability of heavy metal.

The SGH states that plant interactions become less competitive along a gradient of increasing environmental stress^12^. While many studies found supportive evidence for the SGH in various ecosystems^39^, other studies found evidence against it^40^. Therefore, Smit *et al*.^41^ suggested that the SGH should be expanded by considering the characteristics of resource-based versus non-resource-based abiotic stress over wider stress gradients (opposed to low-high contrasts) because they may add complexity not considered in the SGH. Our results supported the SGH, although the relationship between RII and Pb stress level was not a simple linear correlation. The complicated non-linear relations probably resulted from various tolerances, growth performance to Pb stress and AMF dependency among different plant species under Pb stress conditions. In the current study, grass presented a much higher Pb resistance compared to legume plants. The biomass of legume plants decreased with an increase of Pb stress levels when grown individually, while the biomass of grass remained constant regardless of whether it was grown individually or in co-culture. In addition, grass was a much stronger competitor than legume plants and had a competitive advantage in soil nutrient acquisition when grown together with legume plant^15^. The higher competitive ability is likely due to the greater biomass accumulation and faster growth rate compared to legume plants. Therefore, the presence of grass showed a negative neighbor effect on the growth of legume tree at all Pb stress levels. In contrast, the neighbor effects of legume herbs changed from neutral or negative to positive with an increase of Pb stress levels, in consistence with the SGH. These results were supported by a previous study suggesting that the plants growing near N_2_-fixers would receive multiple benefits because N nutrient can be transferred directly via root exudation and mycorrhizal hyphae to neighboring plants^42^.

The present experiment showed a hump-shaped curve in “R + T” and “R + M” planting patterns, while a valley-shaped curve in “R + L” planting pattern coincident with increasing Pb stress level rather than a linear relationship. In a co-culture planting pattern, the legume plants have similar belowground niches and these compatible plants generally have similar requirements for soil nutrients, pH, moisture, etc., resulting in relatively strong competition in benign environments. However, the amelioration of harsh environment by the legume herbs^43^, frequently driven by architecture-mediated protection from stress conditions probably offset resource competition between legume plants at 500 mg kg^−1^ Pb stress level. When the maximum tolerance of the legume herbs growing without neighbors was reached, a reduction of facilitation at the highest Pb stress level (1500 mg kg^−1^) occurred but the net facilitative effects still existed. This sharp reduction of RII can also be partly attributed to high rates of resource uptake by competitors under harsh environmental conditions^15^. However, when the co-cultured grass had much stronger stress tolerance and competitive abilities than target plant (*R. pseudoacacia*), the outcome of the RII was likely to be negative along stress gradients. With an increase of Pb stress level (500 mg kg^−1^), the growth rate of the neighboring grass (*L. perenne*) remained constant but the growth rate of legume tree (*R. pseudoacacia*) was greatly inhibited, thus a stronger negative neighbor effect could be detected.

The present study demonstrates that the effects of AMF on RII depended on the Pb stress level in “R + T” and “R + M” planting patterns. In the control treatment (0 mg kg^−1^), mycorrhizal inoculation markedly increased competition intensity between legume tree and legume herbs. The results were partly consistent with previous study suggesting that the plants that performed better in association with AMF could enhance their own nutrient acquisition and result in growth repression in the neighboring plant^13^,^15^, as reflected in the higher MC and biomass accumulation of *T. pretense* and *M. sativa* compared to *R. pseudoacacia*. At 500 and 1500 mg kg^−1^ Pb stress levels, however, the RII of *T. pratense* and *M. sativa* to *R. pseudoacacia* became positive, and mycorrhizal inoculation decreased the competitive advantages of legume herbs. No differences could be detected between non-mycorrhizal and mycorrhizal treatments, indicating that AMF might play an important role in equalization of co-culturing plants under harsh environment. This phenomenon supports the hypothesis that the plant-plant interactions can be altered by AMF along a stress gradient and also provides us with a better understanding of the roles of AMF in maintaining the diversity of plant population under Pb stress conditions. In contrast, the favorite effects of AMF to legume tree (*R. pseudoacacia*) was not detected in the “R + L” planting pattern, although the legume tree was more AMF-dependent compared to grass (Fig. S1). The results can be partly explained by the competitive advantage that grass had in N and P uptake whereas the legume tree was a weak competitor that presented nutrient use complementarity to grass^15^. In addition, the increase of Pb stress level did not inhibit the growth of grass, and therefore the competitive advantages of grass could persist throughout the whole growing period.

## Conclusions

**Figure 7:**
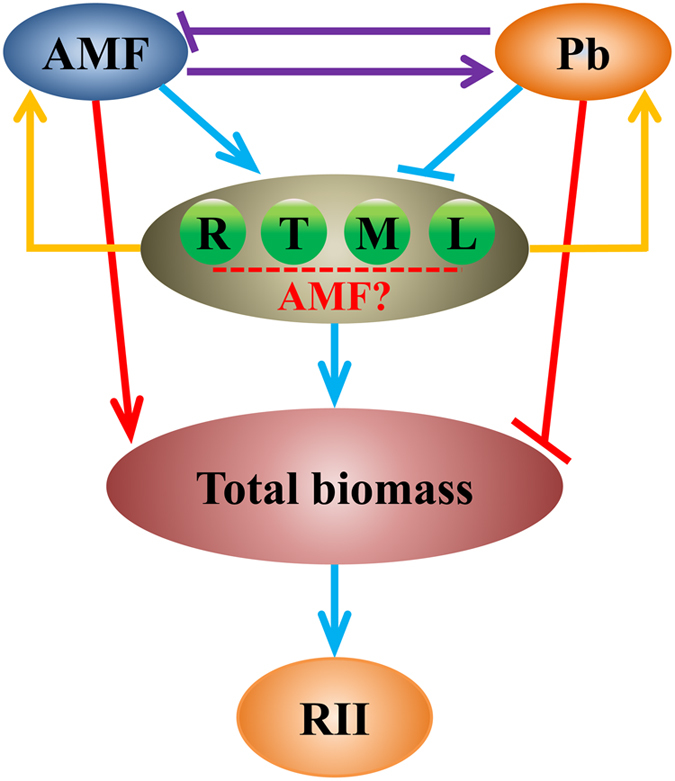
Interactions among AMF, neighbor plants and target plants for phytoremediation under Pb stress conditions. The potential roles of AMF in phytoremediation and tree-herb interactions in Pb contaminated soil. Purple arrows (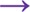): high Pb level inhibits the MC of plants except for *L. perenne* (L), while AMF inoculation significantly increases the root Pb concentration, but decreases the shoot Pb concentration of all plants at the highest Pb level (1500 mg kg^−1^ Pb); Yellow arrows (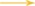): legume and grass neighbors increase and decrease the MC of co-cultured plants, respectively. In addition, legume neighbors improve the Pb accumulation in both shoots and roots of co-cultured plants via reducing soil pH; Red arrows (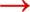): AMF inoculation and Pb addition have positive and negative effects on plant biomass, respectively, and thereby affect the RII and plant completion through a direct pathway; Blue arrows (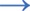): AMF inoculation and Pb addition influence plant photosynthetic parameters, N, P, S and Mg acquisition and allocation. Some of these factors are related to the biomass of plants as shown in Tables S14 and S15. Therefore, AMF can affect RII and plant completion through an indirect pathway. Red dashed line (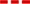): the difference effects of AMF on plant interactions between legume-legume and legume-grass are hypothesized to be resulted from various mycorrhizal dependencies among plants.

The higher root to shoot Pb ratio detected in mycorrhizal plants suggests that mycorrhizal inoculation enhances Pb uptake and accumulation in the root system compared to non-mycorrhizal plants, and *R. intraradices* plays a filtering/sequestering role in Pb detoxification. The presence of legume herbs increased the MC of the target tree (*R. pseudoacacia*), probably due to the common symbiosis signaling pathway activated by both rhizobia and AMF. In addition, legume herbs significantly increased the bioavailability of Pb ions for acquisition via lowering soil pH. Therefore, the legume tree (*R. pseudoacacia*) inoculated with *R. intraradices* and co-cultured with legume herbs can be a promising method to improve the efficiency of phytoremediation. AMF enforced complementary nutrient use between legume-legume and legume-grass probably through the existence of AM fungal hyphal networks between host plants, while the biomass production of plants was closely correlated with N, P, S and Mg concentrations. Thus, the roles of AMF in mediating plant-plant interaction can be attributed to dynamic changes of plant macronutrients caused by mycorrhizal inoculation. The presences of legume herbs and grass mainly showed positive and negative neighbor effects on the legume tree (*R. pseudoacacia*), respectively. However, the relationship between RII and Pb stress levels did not exhibit a simple linear correlation, probably due to various tolerances, competitive abilities and MD among plants. AMF favored legume herbs and amplified plant competition in control treatments, whereas AMF acted to equal the growth of co-cultured plants under Pb stress conditions. A model summarizing the roles of AMF inoculation in plant interactions and phytoremediation is presented in Fig. 7. Our results highlight the important roles of AMF and legume herbs in improving phytoremediation efficiency in Pb polluted soil. The planting pattern of legume tree with deep root systems and long life, together with legume herbs with high MD and the ability to improve Pb accumulation, can be instructive for optimizing in terms of maximizing both phytoremediation efficiency and biomass production. However, further field studies are still required to reveal the important roles of AMF in phytoremediation and ecological stability on larger scales and under more complex environmental conditions.

## Methods

### Culture substrate preparation and Pb addition

The culture substrate consisted of soil and sand (v:v = 1:2). In September 2012, soil was collected from the top layer (0–15 cm) in the Nursery of Forestry College, Northwest A&F University in Yangling, Shaanxi Province, China. The soil was air-dried and passed through 2 mm sieve to remove large stones and plant root debris. The soil is classified as *Eum-Orthric Anthrosols*, with the following properties: pH 7.8 (soil: water = 1:5), 17.4 g kg^−1^ of soil organic matter, 2.18 g kg^−1^ of total nitrogen, 45.4 mg kg^−1^ of available nitrogen, 1.13 g kg^−1^ of total phosphorus, 31.7 mg kg^−1^ of available phosphorus, 179.2 mg kg^−1^ of available potassium, 17.9 mg kg^−1^ of total Pb. The soil was thoroughly mixed with fine sand (2 mm sieve) using a concrete mixer (Croker mixers), and then autoclaved under pressure (0.11 MPa) at 121 °C for 2 h.

One and a half kilograms of air dried culture substrate were filled into plastic pots with a dimension of 14 cm in diameter and 16 cm in height. Pb in the form of lead acetate (Pb(CH_3_COO)_2_·3H_2_O) was amended into the culture substrate through five cycles of Pb-solution-saturation and air-drying processes. Specifically, 150 mg or 450 mg Pb^2+^ was dissolved in 200 mL deionized water before adding to the culture substrate and mixing thoroughly in each pot. Additional deionized water was added to the culture substrate watered to maintain moisture content of 20% on a dry soil basis (around 70% water holding capacity) for three days to allow equilibration. The pots were then left for air drying for four days. The cycle was then repeated four times to achieve a final Pb concentration of 500 mg kg^−1^ or 1500 mg kg^−1^ Pb in culture substrate. Control culture substrate was processed in the same way except for that deionized water without Pb implementation was added in the solution-saturation step. A total of 140 pots were prepared for each of the three treatments, i.e. culture substrate containing no Pb or with Pb in the concentrations of 500 mg kg^−1^ or 1500 mg kg^−1^.

### Plant material and arbuscular mycorrhizal inoculum

Seeds of *R. pseudoacacia* L. were collected from HM polluted site located in Feng County, Shaanxi Province, China (106°39′26′′E, 33°51′21′′N) according to our previous investigation^5^. The seeds were kept in refrigerator at 4 °C before uses. They were surface-sterilized in 10% (v/v) sodium hypochlorite for 8 min, washed eight times with sterile water (floating seeds were discarded), and then pre-germinated on sterile gauze in Petri dishes at 25 °C in the dark. The seeds of clover (*T. pretense*), alfalfa (*M. sativa*) and ryegrass (*L. perenne*) originated from Loess Plateau and were purchased from a local supplier (Yangling, Shaanxi, China). Seeds of red clover (*T. pretense* L.) and alfalfa (*M. sativa* L.) were surface-sterilized in 70% ethanol for 2 min and then in 1% (v/v) sodium hypochlorite for 5 min. After thoroughly rinsing with sterile distilled water, the seeds were germinated on moistened filter paper in Petri dishes at 25 °C in the dark. Seeds of ryegrass (*L. perenne*) were washed in 70% (v/v) ethanol for 2 min, surface sterilized for 8 min in 1% (v/v) sodium hypochlorite, and washed eight times with sterile water. Surface-sterilized seeds were sown on 1.0% (w/v) agar and germinated in the dark at 25 °C. All seeds were germinated in climate chamber (RQH-250, Jing Hong laboratory instrument Co., Ltd. China) for three days, and then transformed into PVC tubes or pots according to the experimental procedure (see sections of Cultivation System and Experimental Set Up).

The AMF species, *Rhizophagus intraradices* (N.C. Schenck & G.S. Sm.) C. Walker & A. Schüßler (BGC BJ09), was provided by Beijing Academy of Agriculture and Forestry Sciences, Beijing, China (http://www.yzs.baafs.net.cn/Default.aspx). The fungus was originally isolated from unpolluted soil and was later propagated in pot culture on maize plants (*Zea mays* L.) grown in pure sand implemented with HMs (Pb and Zn, 1000 mg kg^−1^; Cu, 500 mg kg^−1^; Cd 10 mg kg^−1^) for 14 months in order to acquire HM tolerance. The propagation was allowed to undergo three cycles by continuously planting maize plants in the same pot. Each cycle lasted five months. The colonization and spore density of *R. intraradices* were 94.2% and 589 per 10 g of air-dried sand when plants were harvested. Inoculum from the pot culture comprised a mixture of spores, mycelium, dried sand, and colonized maize root fragments.

### Cultivation system

**Figure 8:**
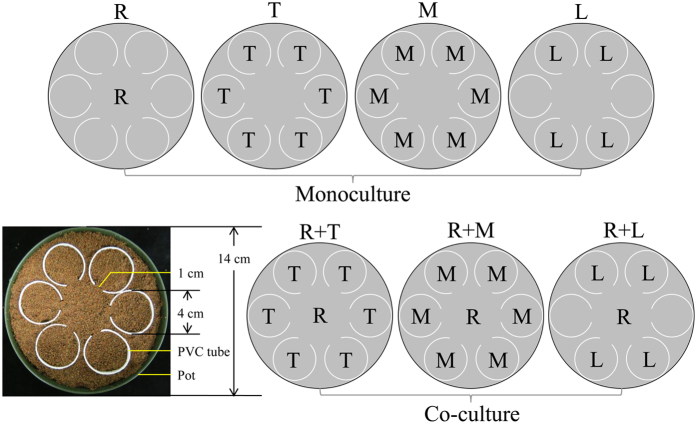
Experimental design. Grey circle (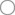) refers to pot; white circle (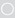) refers to PVC tube. In monocultures, the densities of one *R. pseudoacacia* (R), six *T. pretense* (T) or *M. sativa* (M), or four *L. perenne* (L) were set up per pot; while in co-cultures, one *R. pseudoacacia* was planted with six *T. pretense* in R + T, six *M. sativa* in R + M, or four *L. perenne* in R + L, respectively.

One *R. pseudoacacia* seedling was planted in the center of each pot containing culture substrate. One competitor plant (*T. pretense, M. sativa* or *L. perenne*) was planted in each PVC tube (with a dimension of 4 cm in diameter and 16 cm in height) and then inserted into the surroundings of the *R. pseudoacacia* seedling in pots (Fig. 8). A strip with 1 cm width was cut off from top to bottom of the side of each PVC tube. When inserted in pots, the cut side of PVC tube was arranged facing the center of pots to guide roots growth of competitors from the edge towards the center of pots and to reduce intraspecific competition intensity among competitors. Six *T. pretense* or *M. sativa* plants were cultured per pot (390 plants m^−2^) while four *L. perenne* plants were cultured per pot (260 plants m^−2^), mimicking their respective natural density in local environment. A plastic tray was placed under each pot to collect potential leachate during the experiment.

### Experimental set up

The experiment was conducted in the greenhouse of Northwest A&F University in Shaanxi province of China (34° 15′ 59′′ N, 108° 03′ 39′′ E) from March to May, 2013. The temperature was between 20 to 30 °C and the relative air humidity was 50 to 75%. A 3 × 2 × 7 factorial randomized complete block design was used to present the three Pb pollution levels (Pb0, 0 mg kg^−1^; Pb500, 500 mg kg^−1^; Pb1500, 1500 mg kg^−1^), two AMF treatments (mycorrhizal presence, +AMF; mycorrhizal absence, –AMF) and seven planting patterns, including any monoculture and co-culture planting patterns in individual pots. In monocultures, the densities of one *R. pseudoacacia* (R), six *T. pretense* (T) or *M. sativa* (M), or four *L. perenne* (L) was set up per pot; while in co-cultures, one *R. pseudoacacia* was planted with six *T. pretense* in R + T, six *M. sativa* in R + M, or four *L. perenne* in R + L, respectively (Fig. 8). Ten replicates for each treatment were made, and a total of 420 pots were set up in the experiment. After three days of pre-germination, three seedlings of *T. pretense, M. sativa*, and two seedlings of *L. perenne* were transformed from chamber to each PVC tube in monocultures and co-cultures as shown in Fig. 8. Only one seedling of *R. pseudoacacia* was transformed into the center of each pot because survival rate for *R. pseudoacacia* was almost 100 percent according to our previous experiment^8^. 10 g of AM inoculum were placed 1–2 cm in culture substrate below seedlings. Non-mycorrhizal control plants received 10 mL of the filtered leachate (15 μm) from AM mixed inoculum to correct possible differences in the soil bacterial and non-AMF communities. In order to mimic bacterial community under natural conditions, all pots received a 25 mL washing filtrate (15 μm) from soil collected underneath the *R. pseudoacacia* in Feng County^5^. After eight days of transformation, the plants were thinned to one per PVC tube (Fig. 8) so that each plant species were as homogenous in size as possible after thinning. Plants were irrigated with distilled water to maintain about 70% of water holding capacity according to our previous study^8^, and no additional nutrient was supplied during the whole experiment. Leaf chlorophyll contents and photosynthetic parameters of *R. pseudoacacia* were determined after 78 days thinning; while all plants were harvested after 80 days thinning to determine MC, plant height, plant biomass, Pb concentrations, and macronutrient concentrations.

### Measurements

The net photosynthetic rate (*P*_*n*_), stomatal conductance (*g*_*s*_), intercellular CO_2_ concentration (*C*_*i*_) of *R. pseudoacacia* were measured using a portable open flow gas exchange system LI-6400 (LI-COR, USA) from 9:00 to 11:00 am on the fifth youngest leaf^8^. The photosynthetically active radiation was 1000 ± 17 μmol m^−2^ S^−1^; the leaf temperature was 26.0 ± 1.0 °C; CO_2_ concentration and the ambient water vapor pressure were kept at 418 ± 10 cm^3^ m^−3^ and 1.35 ± 0.10 kPa, respectively. After the photosynthetic parameters have been determined, fresh leaf samples (the fifth leaf) were harvested and cleaned with deionized water to remove any surface contamination. The fresh sample (100 mg) was homogenized in 25 mL acetone (80%) in the dark at room temperature for 10 h. A UV/Vis spectrophotometer (Model Mini 1240 UV-Vis, Shimadzu Corporation, Kyoto, Japan) was used to measure chlorophyll a (Chl *a*) and chlorophyll b (Chl *b*) concentrations at 646 and 663 nm according to Harborne’s method^44^. The leaf Chlorophyll concentrations were calculated according to equations (1) and (2):


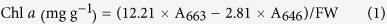



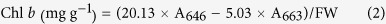


where FW is the leaf fresh weight of *R. pseudoacacia*.

All plants were harvested after 80 days thinning to determine plant height, MC, plant biomass, Pb concentrations, macronutrient concentrations, soil pH and soil diethylene triamine penta acetic acid (DTPA)-extractable Pb concentration. Plant height was measured by using a precision straight edge (Sword fish, China, 0.01 cm). Soil samples were collected from pot center and soil pH was determined using a combination glass electrode (Leici PHS-3D, Shanghai, China) according to the international analysis method of ISO 10390: 2005 (soil:water = 1:5). Soil DTPA-extractable Pb concentration was measured by flame atomic absorption spectrometry (FAAS, Hitachi Z-2000, Tokyo, Japan), following the dissolution of a 0.5 g soil sample with DTPA solution (0.005 M DTPA, 0.01 M CaCl_2_, 0.1 M triethanolamine, pH = 7.3). The shoots of seedlings were cut off and the rest whole pot was washed carefully with tap water to remove soil particles and separate the roots of neighboring seedlings. The root samples were collected and cut into 1 cm length segments. The segments were softened in 2.5% KOH at 90 °C for 1 h and bleached in alkaline hydrogen peroxide at room temperature for 30 min, after which they were acidified in 1% HCl at room temperature for 1 h and stained with trypan blue (0.05%) at 90 °C for 10 min. MC was estimated according to Trouvelot *et al*.^45^. One hundred root fragments per treatment were used to measure the MC under a light microscope (Olympus Bx51, Japan) at 200× magnification. The percentage of mycorrhizal structures in each 1 cm root fragment was assessed as 0, 10, 20 … 100%. The MC was calculated according to equation (3):


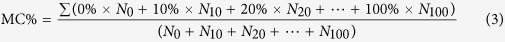


where *N* is the number of root fragments. Plant shoots and roots were dried in an oven at 80 °C for 48 h to determine dry biomass. Na, K, Ca, Mg and Pb concentrations in shoots and roots of plants were determined by Atomic Absorption Spectrophotometer (Hitachi Z-2000, Tokyo, Japan) using different lamps after wet digestion of dried and ground sub-samples in a HNO_3_-HClO_4_ (4:1) acid mixture. Turbidimetry was used to determine total S content according to the description of Tabatabai and Bremner^46^. The Kjeldahl method^47^ was used to determine the total N content and the vanadomolybdate method^48^ was used to calculate total P content in shoot and root samples of plants. All analyses were carried out in quadruplicate.

### Statistical analysis

The TF indicates the efficiency of a plant translocate the metal from its root to shoot^2^. It is calculated according to equation (4):


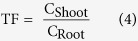


where C_Shoot_ and C_Root_ are the Pb concentrations in plant shoot and root, respectively.

The relative competition intensity of neighbor effect was calculated by using RII, which is defined as equation (5)^49^:


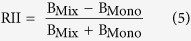


where B_Mix_ is the total biomass of the target plant with neighbor, and B_Mono_ is the total biomass of the target plant without neighbor. The value of RII is limited between −1 and +1. Furthermore, positive values indicate that the neighbors present positive effects (facilitation) on target plant, while negative values indicate that the neighbors present negative effects (competition) on target plant.

The MD presents the degree to which a plant relies upon the mycorrhizal condition to produce its maximum growth at a given level of soil fertility^50^. It is calculated according to equation (6):


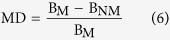


where B_M_ is the total biomass of mycorrhizal plants and B_NM_ is the total biomass of non-mycorrhizal plants.

Prior to data analysis, the Kolmogorov-Smirnov test was used to check for data normality and the Levene test for homogeneity of variance in SPSS 16.0 for Windows 7 (SPSS Inc., Chicago, IL, USA). In the present study, all the original datasets conformed to a normal distribution. When necessary, dependent variables were transformed using the natural logarithmic, arcsine or Box-Cox functions to achieve requirements of homogeneity of variance (*P* > 0.05). Potential differences among different treatments were analyzed using two-way and three-way ANOVAs followed by Duncan’s multiple range tests. Two-way ANOVA was used to determine the significance of the effects of AMF inoculation, Pb level and their interactions (AMF × Pb) on plant height, plant dry weight, macronutrient concentrations, photosynthetic parameters, and RII in the same planting pattern. Three-way ANOVA was performed to determine the significance of the effects of neighbor presence, AMF inoculation, Pb level, and their interactions (neighbor × AMF, neighbor × Pb, AMF × Pb, and neighbor × AMF × Pb) on plant height, plant dry weight, Pb concentrations, macronutrient concentrations, and photosynthetic parameters among different planting patterns. Bivariate relationships were conducted using Pearson correlation and the associations among plant biomass, macronutrient concentrations, and photosynthetic parameter variables in the same planting pattern were performed using multiple linear regressions. Pearson correlation coefficients were used to determine the relationships among plant growth parameters and physiological parameters. All tests were two-tailed and significance of the obtained results was judged at the 5% level. All data in the figures and tables are presented with original data, and they are presented as mean ± SD (standard deviation).

## Additional Information

**How to cite this article**: Yang, Y. *et al*. The roles of arbuscular mycorrhizal fungi (AMF) in phytoremediation and tree-herb interactions in Pb contaminated soil. *Sci. Rep.* **6**, 20469; doi: 10.1038/srep20469 (2016).

## Supplementary Material

Supplementary Information

## Figures and Tables

**Figure 1:**
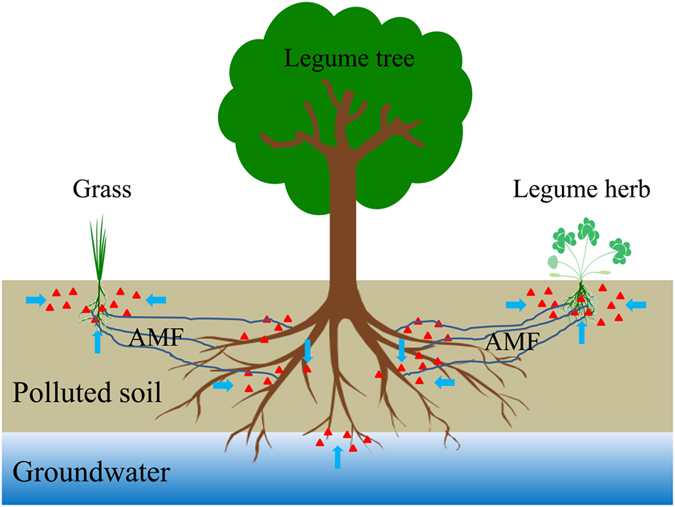
Phytoremediation technology in a three-dimensional scale can be used for remediating HM polluted soil and groundwater. In the shallow soil layer, the HMs can be extracted and/or immobilized by herbaceous plants with shallow root systems. However, in the deep soil layer, the HMs can be extracted and/or stabilized by tree species with deep root systems to prevent soil and groundwater pollution. The red up-triangles (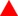) represent the HM pollutant.
