# Supplementary material for: The roles of arbuscular mycorrhizal fungi (AMF) in phytoremediation and tree-herb interactions in Pb contaminated soil
Source: Sci Rep. 2016 Feb 4;6:20469. doi: 10.1038/srep20469 (PMC4740888; doi:10.1038/srep20469)
Supplement: Supplementary Information [file srep20469-s1.doc]

**Supplementary materials**

The roles of arbuscular mycorrhizal fungi (AMF) in phytoremediation and tree-herb interactions in Pb contaminated soil

Yurong Yang^1,2¶^, Yan Liang^3,4¶^, Xiaozhen Han^5^, Tsan-Yu Chiu^3,4^, Amit Ghosh^6^, Hui Chen^2^* & Ming Tang^2^*

^1^State Key Laboratory of Soil Erosion and Dryland Farming on the Loess Plateau, Northwest A&F University, Yangling, Shaanxi 712100, China.

^2^College of Forestry, Northwest A&F University, Yangling, Shaanxi 712100, China.

^3^Joint BioEnergy Institute, 5885 Hollis St, Emeryville, CA 94608, USA.

^4^Physical Biosciences Division, Lawrence Berkeley National Laboratory, Berkeley, CA 94720, USA.

^5^Plant Systems Biology Lab, Botany and Plant Science, School of Natural Sciences, National University of Ireland, Galway, Ireland.

^6^School of Energy Science and Engineering, PK Sinha Centre for Bioenergy, Indian Institute of Technology Kharagpur, Kharagpur 721302, India.

^¶^These authors contributed equally to this work.

***Corresponding authors:**

[chenhui@nwsuaf.edu.cn](mailto:chenhui@nwsuaf.edu.cn); Tel.: +86 029 87082083 (H.C.)

[tangm@nwsuaf.edu.cn](mailto:tangm@nwsuaf.edu.cn); Tel.: +86 029 87080807 (M.T.)

**Supplementary materials**

**Table S1.** The MC, plant height, root dry weight, shoot dry weight, total dry weight and root/shoot ratios of plants in different treatments. Values are means ± SD of four replicates. Monocultures of *R. pseudoacacia*, *T. pretense*, *M. sativa* and *L. perenne* are represented by R, T, M and L, respectively. Co-culture planting pattern is represented by the two co-culture species connected with the plus sign (+). The results are reported as the mean (n = 4) ± SD. Different letters indicate that significant differences were detected in these parameters of each plant species grown in one planting pattern but at different Pb levels and AMF inoculation status by Duncan's multiple-range tests (*P* < 0.05).

| Plant  species | Planting  pattern | Pb  level | MC  (%) | Plant height  (cm) | | Dry weight (g pot^-1^) | | | | | | | |
| --- | --- | --- | --- | --- | --- | --- | --- | --- | --- | --- | --- | --- | --- |
|  |  |  |  |  |  | Root | | Shoot | | Total | | Root/shoot | |
|  |  |  |  | -AMF | +AMF | -AMF | +AMF | -AMF | +AMF | -AMF | +AMF | -AMF | +AMF |
| R | R | Pb0 | 41.0±3.34bc | 35.3±4.29b | 42.3±2.47a | 1.09±0.22a | 1.21±0.08a | 4.37±0.25c | 5.24±0.26a | 5.46±0.22c | 6.45±0.21a | 0.25±0.06a | 0.23±0.02a |
|  |  | Pb500 | 34.2±2.27de | 29.1±3.92cd | 39.1±3.02ab | 1.03±0.18a | 1.04±0.12a | 3.84±0.21d | 4.84±0.22b | 4.87±0.06d | 5.88±0.30b | 0.27±0.06a | 0.22±0.02a |
|  |  | Pb1500 | 30.0±2.89e | 24.7±2.59d | 30.4±1.92c | 0.76±0.12b | 0.80±0.14b | 2.82±0.15e | 3.79±0.27d | 3.58±0.14e | 4.59±0.39d | 0.27±0.05a | 0.21±0.03a |
|  | R+T | Pb0 | 46.9±5.44a | 32.9±1.37b | 40.7±2.54a | 0.97±0.09b | 1.07±0.10ab | 4.53±0.06c | 4.86±0.24b | 5.51±0.14c | 5.93±0.25b | 0.21±0.02a | 0.22±0.02a |
|  |  | Pb500 | 45.9±2.73ab | 32.8±1.37b | 40.8±2.32a | 1.09±0.06a | 1.16±0.06a | 4.56±0.16c | 5.36±0.13a | 5.65±0.21c | 6.52±0.11a | 0.24±0.01a | 0.22±0.01a |
|  |  | Pb1500 | 36.1±3.85cd | 25.5±1.43c | 32.5±2.06b | 0.71±0.06c | 0.82±0.04c | 3.03±0.15e | 3.75±0.13d | 3.74±0.21e | 4.57±0.10d | 0.23±0.01a | 0.22±0.02a |
|  | R+M | Pb0 | 47.2±3.22a | 31.5±1.98b | 41.7±1.79a | 1.09±0.04a | 0.93±0.09b | 4.18±0.21c | 4.63±0.31b | 5.27±0.25b | 5.56±0.35b | 0.26±0.01ab | 0.20±0.02c |
|  |  | Pb500 | 45.0±4.12ab | 32.3±1.37b | 40.1±1.55a | 1.05±0.11a | 1.05±0.09a | 4.52±0.20bc | 5.30±0.26a | 5.57±0.10b | 6.35±0.21a | 0.23±0.03bc | 0.20±0.02c |
|  |  | Pb1500 | 38.7±3.20cd | 24.1±2.22c | 31.3±1.35b | 0.79±0.03c | 0.84±0.07bc | 2.89±0.20e | 3.81±0.19d | 3.68±0.19d | 4.65±0.25c | 0.27±0.02a | 0.22±0.01c |
|  | R+L | Pb0 | 30.5±3.15e | 25.5±2.82cd | 34.1±1.96a | 0.94±0.12ab | 0.98±0.06a | 3.67±0.11b | 4.43±0.07a | 4.61±0.19b | 5.41±0.06a | 0.26±0.03ab | 0.22±0.02b |
|  |  | Pb500 | 30.0±2.86e | 22.3±2.01d | 30.0±2.50b | 0.72±0.08de | 0.82±0.07cd | 2.95±0.10d | 3.48±0.06c | 3.67±0.09d | 4.29±0.11c | 0.24±0.03ab | 0.24±0.02ab |
|  |  | Pb1500 | 29.4±3.75e | 22.9±1.48d | 28.6±b1.83c | 0.69±0.08e | 0.85±0.05bc | 2.61±0.15e | 3.39±0.13c | 3.31±0.15e | 4.24±0.16c | 0.27±0.02a | 0.25±0.01ab |
| T | T | Pb0 | 48.5±1.79b | - | - | 0.33±0.04a | 0.38±0.03a | 0.62±0.05ab | 0.77±0.05a | 0.95±0.05bc | 1.16±0.08a | 0.53±0.09b | 0.49±0.01b |
|  |  | Pb500 | 47.9±6.64b | - | - | 0.31±0.02ab | 0.32±0.01bc | 0.57±0.04bc | 0.74±0.09a | 0.88±0.05c | 1.06±0.11ab | 0.54±0.06b | 0.44±0.04b |
|  |  | Pb1500 | 36.6±1.94c | - | - | 0.25±0.03c | 0.26±0.03c | 0.40±0.04d | 0.61±0.06c | 0.59±0.05d | 0.87±0.08c | 0.64±0.10a | 0.43±0.05b |
|  | R+T | Pb0 | 57.0±3.52a | - | - | 0.30±0.02a | 0.33±0.03b | 0.64±0.04a | 0.72±0.02a | 0.94±0.04b | 1.04±0.05a | 0.48±0.05b | 0.45±0.03b |
|  |  | Pb500 | 52.9±2.45ab | - | - | 0.30±0.02a | 0.30±0.01ab | 0.62±0.06a | 0.68±0.01a | 0.93±0.06b | 0.98±0.02b | 0.48±0.04b | 0.44±0.02b |
|  |  | Pb1500 | 45.9±7.09b | - | - | 0.28±0.01b | 0.27±0.01b | 0.50±0.03a | 0.58±0.04b | 0.78±0.04d | 0.86±0.03c | 0.55±0.03a | 0.47±0.05b |
| M | M | Pb0 | 50.0±3.20bc | - | - | 0.31±0.02a | 0.35±0.04a | 0.61±0.04ab | 0.73±0.04ab | 0.92±0.02b | 1.07±0.07a | 0.50±0.05ab | 0.48±0.03ab |
|  |  | Pb500 | 45.5±4.94cd | - | - | 0.29±0.02a | 0.32±0.03a | 0.59±0.03b | 0.70±0.03a | 0.89±0.05b | 1.03±0.06a | 0.50±0.03ab | 0.46±0.03b |
|  |  | Pb1500 | 35.8±3.35e | - | - | 0.25±0.01b | 0.30±0.02b | 0.47±0.04d | 0.62±0.03c | 0.72±0.04c | 0.92±0.05b | 0.53±0.05a | 0.47±0.03ab |
|  | R+M | Pb0 | 58.8±2.63a | - | - | 0.29±0.02a | 0.29±0.01ab | 0.59±0.02c | 0.70±0.02b | 0.88±0.04bc | 0.99±0.03a | 0.49±0.02a | 0.41±0.01b |
|  |  | Pb500 | 51.7±4.46b | - | - | 0.29±0.01a | 0.29±0.01ab | 0.63±0.03b | 0.69±0.03a | 0.92±0.04b | 0.98±0.03a | 0.46±0.01ab | 0.41±0.04b |
|  |  | Pb1500 | 43.4±2.46d | - | - | 0.26±0.01b | 0.25±0.01c | 0.53±0.03d | 0.59±0.03bc | 0.79±0.02d | 0.85±0.03c | 0.49±0.04a | 0.43±0.03b |
| L | L | Pb0 | 21.7±2.50b | - | - | 1.75±0.15a | 1.76±0.17a | 2.62±0.15abc | 2.78±0.13a | 4.38±0.26a | 4.54±0.28a | 0.67±0.05a | 0.63±0.05ab |
|  |  | Pb500 | 20.4±2.12b | - | - | 1.76±0.14a | 1.70±0.06a | 2.57±0.14bc | 2.49±0.10c | 4.33±0.26a | 4.19±0.15a | 0.69±0.04a | 0.68±0.01a |
|  |  | Pb1500 | 23.4±2.21b | - | - | 1.59±0.13a | 1.59±0.13a | 2.79±0.15a | 2.71±0.07ab | 4.38±0.25a | 4.30±0.14a | 0.57±0.03b | 0.59±0.05b |
|  | R+L | Pb0 | 29.3±1.40a | - | - | 2.10±0.14ab | 2.22±0.15a | 3.16±0.08cd | 3.83±0.14b | 5.26±0.18b | 6.05±0.29a | 0.66±0.04a | 0.58±0.02bc |
|  |  | Pb500 | 27.1±2.25a | - | - | 2.04±0.12ab | 2.12±0.06ab | 3.02±0.12d | 3.83±0.05b | 5.06±0.17b | 5.95±0.03a | 0.68±0.05a | 0.55±0.02cd |
|  |  | Pb1500 | 26.9±1.88a | - | - | 2.01±0.13b | 2.08±0.04ab | 3.20±0.14c | 4.06±0.11a | 5.21±0.10b | 6.14±0.09a | 0.63±0.06ab | 0.51±0.02d |

**Table S2.** Multiple ANOVA comparisons of MC, plant height, root dry weight, shoot dry weight, total dry weight and root/shoot ratios of *R. pseudoacacia* affected by neighbor plant (neighbor), AMF inoculation (AMF), Pb level (Pb) and their interactions in different treatments. Monocultures of *R. pseudoacacia*, *T. pretense*, *M. sativa* and *L. perenne* are represented by R, T, M and L, respectively. Co-culture planting pattern is represented by the two co-culture species connected with the plus sign (+). (** *P* < 0.01; * *P* < 0.05; ns, no significance).

| Planting  pattern | Significance | MC | | Height | | Dry weight | | | | | | Root/shoot | |
| --- | --- | --- | --- | --- | --- | --- | --- | --- | --- | --- | --- | --- | --- |
|  |  |  |  |  |  | Root | | Shoot | | Total | |  |  |
|  |  | *F* | *P* | *F* | *P* | *F* | *P* | *F* | *P* | *F* | *P* | *F* | *P* |
| R | AMF | - | - | 34.8 | 0.00** | 0.85 | 0.37ns | 99.6 | 0.00** | 100 | 0.00** | 6.10 | 0.02* |
|  | Pb | 15.1 | 0.00** | 25.8 | 0.00** | 12.7 | 0.00** | 87.3 | 0.00** | 121 | 0.00** | 0.01 | 1.00ns |
|  | AMF × Pb | - | - | 0.97 | 0.40ns | 0.25 | 0.78ns | 0.18 | 0.84ns | 0.01 | 0.99ns | 0.48 | 0.63ns |
| R+T | AMF | - | - | 94.6 | 0.00** | 9.36 | 0.007** | 94.0 | 0.00** | 93.9 | 0.00** | 2.90 | 0.11ns |
|  | Pb | 8.17 | 0.01** | 44.5 | 0.00** | 53.7 | 0.00** | 232 | 0.00** | 262 | 0.00** | 1.07 | 0.36ns |
|  | AMF × Pb | - | - | 0.19 | 0.83ns | 0.15 | 0.87ns | 5.29 | 0.02* | 3.75 | 0.04* | 1.80 | 0.19ns |
| Neighbor | | 29.2 | 0.00** | 0.91 | 0.35ns | 0.27 | 0.61ns | 12.1 | 0.00** | 8.50 | 0.01** | 3.23 | 0.08ns |
| AMF | | - | - | 105 | 0.00** | 4.60 | 0.04* | 187 | 0.00** | 189 | 0.00** | 8.42 | 0.01** |
| Pb | | 19.1 | 0.00** | 59.8 | 0.00** | 36.8 | 0.00** | 252 | 0.00** | 319 | 0.00** | 0.13 | 0.88ns |
| Neighbor × AMF | | - | - | 0.00 | 1.00ns | 0.23 | 0.64ns | 8.34 | 0.01** | 5.72 | 0.02* | 3.04 | 0.09ns |
| Neighbor × Pb | | 1.71 | 0.21ns | 3.64 | 0.04* | 3.51 | 0.04* | 14.3 | 0.00** | 20.0 | 0.00** | 0.12 | 0.88ns |
| AMF × Pb | | - | - | 1.09 | 0.35ns | 0.31 | 0.74ns | 2.65 | 0.08ns | 1.46 | 0.25ns | 1.19 | 0.32ns |
| Neighbor × AMF × Pb | | - | - | 0.91 | 0.35ns | 0.15 | 0.86ns | 0.87 | 0.43ns | 1.13 | 0.33ns | 0.08 | 0.92ns |
|  | |  |  |  |  |  |  |  |  |  |  |  |  |
| R+M | AMF | - | - | 101 | 0.00** | 1.31 | 0.27ns | 56.4 | 0.00** | 48.6 | 0.00** | 30.7 | 0.00** |
|  | Pb | 6.12 | 0.02* | 38.9 | 0.00** | 21.4 | 0.00** | 94.0 | 0.00** | 120 | 0.00** | 4.25 | 0.03* |
|  | AMF × Pb | - | - | 0.10 | 0.91ns | 4.17 | 0.03* | 2.09 | 0.15ns | 4.30 | 0.03* | 0.74 | 0.49ns |
| Neighbor | | 42.4 | 0.00** | 0.04 | 0.84ns | 0.75 | 0.39ns | 1.17 | 0.29ns | 0.37 | 0.55ns | 1.01 | 0.32ns |
| AMF | | - | - | 112 | 0.00** | 0.09 | 0.76ns | 153 | 0.00** | 144 | 0.00** | 21.4 | 0.00** |
| Pb | | 18.3 | 0.00** | 56.7 | 0.00** | 26.3 | 0.00** | 164 | 0.00** | 219 | 0.00** | 0.70 | 0.51ns |
| Neighbor × AMF | | - | - | 0.52 | 0.48ns | 1.79 | 0.19ns | 3.00 | 0.09ns | 5.40 | 0.03* | 0.03 | 0.86ns |
| Neighbor × Pb | | 1.08 | 0.36ns | 3.90 | 0.03* | 2.63 | 0.09ns | 17.5 | 0.00** | 21.7 | 0.00** | 0.91 | 0.41ns |
| AMF × Pb | | - | - | 0.67 | 0.52ns | 0.31 | 0.74ns | 1.67 | 0.20ns | 2.24 | 0.12ns | 0.26 | 0.77ns |
| Neighbor × AMF × Pb | | - | - | 0.04 | 0.84ns | 0.79 | 0.18ns | 0.60 | 0.56ns | 1.90 | 0.16ns | 0.80 | 0.46ns |
|  | |  |  |  |  |  |  |  |  |  |  |  |  |
| R+L | AMF | - | - | 70.2 | 0.00** | 10.4 | 0.005** | 237 | 0.00** | 208 | 0.00** | 4.38 | 0.05ns |
|  | Pb | 0.12 | 0.89ns | 8.62 | 0.00** | 18.4 | 0.00** | 205 | 0.00** | 198 | 0.00** | 1.61 | 0.23ns |
|  | AMF × Pb | - | - | 0.94 | 0.41ns | 1.20 | 0.33ns | 3.33 | 0.06ns | 2.73 | 0.09ns | 0.64 | 0.54ns |
| Neighbor | | 16.43 | 0.00** | 46.0 | 0.00** | 19.9 | 0.00** | 193 | 0.00** | 238 | 0.00** | 0.19 | 0.66ns |
| AMF | | - | - | 66.6 | 0.00** | 5.07 | 0.03* | 243 | 0.00** | 244 | 0.00** | 9.95 | 0.00** |
| Pb | | 7.94 | 0.00** | 94.2 | 0.00** | 22.7 | 0.00** | 197 | 0.00** | 246 | 0.00** | 0.28 | 0.76ns |
| Neighbor × AMF | | - | - | 33.1 | 0.00** | 0.36 | 0.55ns | 6.19 | 0.02* | 3.70 | 0.06ns | 1.52 | 0.23ns |
| Neighbor × Pb | | 5.30 | 0.02* | 0.03 | 0.87ns | 4.90 | 0.01* | 20.6 | 0.00** | 30.0 | 0.00** | 0.41 | 0.67ns |
| AMF × Pb | | - | - | 8.56 | 0.00** | 0.11 | 0.90ns | 0.40 | 0.68ns | 0.60 | 0.56ns | 0.10 | 0.91ns |
| Neighbor × AMF × Pb | | - | - | 1.39 | 0.26ns | 0.75 | 0.48ns | 1.10 | 0.35ns | 0.65 | 0.53ns | 0.93 | 0.40ns |

**Table S3.** Multiple ANOVA comparisons of MC, plant height, root dry weight, shoot dry weight, total dry weight and root/shoot ratios of *T. pretense*, *M. sativa* and *L. perenne* affected neighbor plant (neighbor), AMF inoculation (AMF), Pb level (Pb) and their interactions in different treatments. Monoculture of *R. pseudoacacia*, *T. pretense*, *M. sativa* and *L. perenne* are represented by R, T, M and L, respectively. Co-culture planting pattern is represented by the two co-culture species connected with the plus sign (+). (** *P* < 0.01; * *P* < 0.05; ns, no significance).

| Plant  species | Planting  pattern | Significance | MC | | Dry weight | | | | | | | |
| --- | --- | --- | --- | --- | --- | --- | --- | --- | --- | --- | --- | --- |
|  |  |  |  |  | Root | | Shoot | | Total | | Root/shoot | |
|  |  |  | *F* | *P* | *F* | *P* | *F* | *P* | *F* | *P* | *F* | *P* |
| T | T | AMF | - | - | 4.37 | 0.05ns | 52.4 | 0.00** | 44.3 | 0.00** | 18.7 | 0.00** |
|  |  | Pb | 10.5 | 0.00** | 21.8 | 0.00** | 23.8 | 0.00** | 33.3 | 0.00** | 1.13 | 0.35ns |
|  |  | AMF × Pb | - | - | 1.37 | 0.28ns | 0.47 | 0.63ns | 0.12 | 0.89ns | 3.47 | 0.05ns |
|  | R+T | AMF | - | - | 0.84 | 0.37ns | 23.0 | 0.00** | 20.9 | 0.00** | 9.91 | 0.006** |
|  |  | Pb | 5.48 | 0.03* | 11.0 | 0.00** | 30.9 | 0.00** | 38.4 | 0.00** | 4.41 | 0.03* |
|  |  | AMF × Pb | - | - | 1.43 | 0.27ns | 0.35 | 0.71ns | 0.75 | 0.49ns | 1.10 | 0.36ns |
|  | Neighbor | | 17.5 | 0.00** | 3.57 | 0.07ns | 0.09 | 0.77* | 0.28 | 0.60ns | 4.57 | 0.04* |
|  | AMF | | - | - | 5.15 | 0.03* | 75.3 | 0.00** | 64.8 | 0.00** | 28.3 | 0.00** |
|  | Pb | | 14.7 | 0.00** | 32.4 | 0.00** | 50.2 | 0.00** | 65.3 | 0.00** | 3.64 | 0.04* |
|  | Neighbor × AMF | | - | - | 1.84 | 0.18ns | 12.9 | 0.00* | 12.3 | 0.00** | 4.62 | 0.04* |
|  | Neighbor × Pb | | 0.53 | 0.60ns | 5.80 | 0.007** | 1.44 | 0.25ns | 3.80 | 0.03* | 0.27 | 0.76ns |
|  | AMF × Pb | | - | - | 2.58 | 0.09ns | 0.54 | 0.59ns | 0.46 | 0.64ns | 4.57 | 0.02* |
|  | Neighbor × AMF × Pb | | - | - | 0.19 | 0.83ns | 0.33 | 0.72ns | 0.09 | 0.92ns | 1.17 | 0.32ns |
|  |  | |  |  |  |  |  |  |  |  |  |  |
| M | M | AMF | - | - | 0.74 | 0.40ns | 13.3 | 0.00** | 60.3 | 0.00** | 6.12 | 0.02* |
|  |  | Pb | 13.8 | 0.00** | 26.4 | 0.00** | 32.4 | 0.00** | 26.1 | 0.00** | 0.86 | 0.44ns |
|  |  | AMF × Pb | - | - | 0.83 | 0.45ns | 0.32 | 0.73ns | 0.79 | 0.47ns | 0.26 | 0.77ns |
|  | R+M | AMF | - | - | 4.72 | 0.04* | 27.9 | 0.00** | 34.3 | 0.00** | 29.7 | 0.00** |
|  |  | Pb | 21.8 | 0.00** | 15.2 | 0.00** | 27.1 | 0.00** | 44.6 | 0.00** | 1.49 | 0.25ns |
|  |  | AMF × Pb | - | - | 0.70 | 0.51ns | 0.03 | 0.97ns | 1.78 | 0.20ns | 0.78 | 0.47ns |
|  | Neighbor | | 26.0 | 0.00** | 4.87 | 0.03* | 6.76 | 0.013* | 2.84 | 0.10ns | 17.6 | 0.00** |
|  | AMF | | - | - | 4.54 | 0.04* | 37.5 | 0.00** | 93.7 | 0.00** | 26.2 | 0.00** |
|  | Pb | | 34.0 | 0.00** | 40.9 | 0.00** | 53.0 | 0.00** | 60.1 | 0.00** | 2.06 | 0.14ns |
|  | Neighbor × AMF | | - | - | 0.81 | 0.38ns | 0.13 | 0.72ns | 12.8 | 0.00** | 1.07 | 0.31ns |
|  | Neighbor × Pb | | 0.26 | 0.77ns | 1.00 | 0.38ns | 7.81 | 0.00** | 2.05 | 0.14ns | 0.07 | 0.94ns |
|  | AMF × Pb | | - | - | 0.11 | 0.90ns | 0.30 | 0.75ns | 0.84 | 0.44ns | 0.24 | 0.79ns |
|  | Neighbor × AMF × Pb | | - | - | 1.42 | 0.26ns | 0.13 | 0.88ns | 1.28 | 0.29ns | 0.61 | 0.55ns |
|  |  | |  |  |  |  |  |  |  |  |  |  |
| L | L | AMF | - | - | 0.12 | 0.73ns | 0.00 | 0.96ns | 0.03 | 0.86ns | 0.25 | 0.62ns |
|  |  | Pb | 1.72 | 0.23ns | 3.40 | 0.06ns | 6.61 | 0.007** | 1.50 | 0.25ns | 12.9 | 0.00** |
|  |  | AMF × Pb | - | - | 0.16 | 0.85ns | 2.32 | 0.13ns | 0.97 | 0.40ns | 0.87 | 0.44ns |
|  | R+L | AMF | - | - | 3.72 | 0.07ns | 301 | 0.00** | 169 | 0.00** | 42.8 | 0.00** |
|  |  | Pb | 2.06 | 0.18ns | 2.10 | 0.15ns | 7.21 | 0.01** | 2.64 | 0.10ns | 3.72 | 0.05* |
|  |  | AMF × Pb | - | - | 0.14 | 0.87ns | 1.53 | 0.24ns | 0.33 | 0.73ns | 0.53 | 0.60ns |
|  | Neighbor | | 48.8 | 0.00** | 123 | 0.00** | 626 | 0.00** | 478 | 0.00** | 8.59 | 0.01** |
|  | AMF | | - | - | 1.00 | 0.32ns | 131 | 0.00** | 55.0 | 0.00** | 24.0 | 0.00** |
|  | Pb | | 1.67 | 0.22ns | 5.00 | 0.01* | 12.8 | 0.00** | 3.28 | 0.05* | 14.7 | 0.00** |
|  | Neighbor × AMF | | - | - | 2.34 | 0.14ns | 129 | 0.00** | 59.3 | 0.00** | 17.5 | 0.00** |
|  | Neighbor × Pb | | 2.05 | 0.16ns | 0.69 | 0.51ns | 0.89 | 0.42ns | 0.49 | 0.62ns | 2.28 | 0.12ns |
|  | AMF × Pb | | - | - | 0.20 | 0.82ns | 0.17 | 0.84ns | 0.28 | 0.76ns | 0.11 | 0.90ns |
|  | Neighbor × AMF × Pb | | - | - | 0.11 | 0.90ns | 3.79 | 0.03* | 1.22 | 0.31ns | 1.30 | 0.29ns |

**Table S4.** Macronutrient concentrations in the shoots and roots of *R. pseudoacacia* in different treatments. Values are means ± SD of four replicates. Monocultures of *R. pseudoacacia*, *T. pretense*, *M. sativa* and *L. perenne* are represented by R, T, M and L, respectively. Co-culture planting pattern is represented by the two co-culture species connected with the plus sign (+). The results are reported as the mean (n = 4) ± SD. Different letters indicate that significant differences were detected in macronutrient concentrations of each plant species grown in one planting pattern but at different Pb levels and AMF inoculation status by Duncan's multiple-range tests (*P* < 0.05).

| Planting  pattern | AMF | Pb  level | Macronutrient concentration | | | | | | | | | | | | | |
| --- | --- | --- | --- | --- | --- | --- | --- | --- | --- | --- | --- | --- | --- | --- | --- | --- |
|  |  |  | N (g kg^-1^) | | P (g kg^-1^) | | S (g kg^-1^) | | Na (mg kg^-1^) | | K (g kg^-1^) | | Ca (g kg^-1^) | | Mg (g kg^-1^) | |
|  |  |  | Shoot | Root | Shoot | Root | Shoot | Root | Shoot | Root | Shoot | Root | Shoot | Root | Shoot | Root |
| R | -AMF | Pb0 | 19.6±1.64b | 14.2±1.74a | 1.29±0.17c | 0.79±0.04b | 2.26±0.09a | 1.92±0.14a | 98.0±16.6a | 90.7±16.8a | 15.7±4.21a | 10.5±2.99a | 10.3±2.82a | 12.9±1.98a | 2.05±0.19b | 2.56±0.19ab |
|  |  | Pb500 | 15.8±1.57c | 11.9±1.03b | 0.96±0.11d | 0.64±0.04c | 1.95±0.08b | 1.56±0.09c | 103±15.3a | 103±20.8a | 15.3±2.47a | 10.3±1.95a | 10.9±1.24a | 13.5±2.62a | 2.02±0.21b | 2.30±0.12b |
|  |  | Pb1500 | 12.5±1.09d | 9.71±1.29c | 0.66±0.06e | 0.50±0.03d | 1.60±0.07c | 1.22±0.09d | 94.9±23.0a | 86.7±21.9a | 16.9±4.50a | 10.3±2.41a | 12.7±2.58a | 13.3±2.57a | 1.58±0.13c | 2.00±0.16c |
|  | +AMF | Pb0 | 22.1±1.66a | 15.1±0.85a | 1.92±0.14a | 1.09±0.06a | 2.32±0.14a | 1.82±0.09ab | 96.2±19.6a | 98.2±15.9a | 16.7±4.50a | 9.18±2.50a | 11.8±2.18a | 12.9±2.47a | 2.60±0.23a | 2.80±0.23a |
|  |  | Pb500 | 19.7±1.38b | 13.6±0.67ab | 1.61±0.14b | 0.99±0.13a | 2.32±0.17a | 1.71±0.06b | 99.2±14.0a | 87.3±15.1a | 17.8±4.71a | 10.1±2.31a | 11.2±3.01a | 13.8±2.21a | 2.51±0.25a | 2.60±0.12a |
|  |  | Pb1500 | 16.7±1.12c | 12.1±0.76b | 1.26±0.09c | 0.81±0.06b | 1.87±0.11b | 1.42±0.10c | 94.0±15.3a | 82.9±15.7a | 15.7±3.62a | 8.79±3.32a | 12.3±2.06a | 13.8±2.23a | 2.32±0.23ab | 2.30±0.20b |
| R+T | -AMF | Pb0 | 21.9±1.06b | 14.4±1.31bc | 1.31±0.06c | 0.79±0.06cd | 2.27±0.10a | 1.81±0.13a | 105±15.5a | 94.4±18.6a | 16.4±2.85a | 9.47±2.84a | 10.8±2.79a | 12.3±1.62a | 1.95±0.16cd | 2.48±0.27b |
|  |  | Pb500 | 18.5±1.13c | 13.9±1.08bc | 1.16±0.10d | 0.69±0.03d | 2.04±0.18b | 1.51±0.06bc | 96.2±16.1a | 84.2±15.4a | 15.6±3.41a | 9.26±2.54a | 12.2±2.41a | 12.9±1.39a | 2.11±0.12c | 2.40±0.16b |
|  |  | Pb1500 | 14.7±1.12d | 11.5±1.17d | 0.83±0.04e | 0.52±0.03e | 1.74±0.07c | 1.39±0.15c | 102±15.6a | 91.3±16.5a | 17.4±3.57a | 10.1±2.61a | 11.8±2.82a | 12.2±2.57a | 1.84±0.10d | 2.03±0.33c |
|  | +AMF | Pb0 | 24.4±1.02a | 17.1±1.59a | 2.11±0.14a | 1.37±0.06a | 2.27±0.06a | 1.97±0.14a | 97.2±18.4a | 89.7±21.9a | 15.0±4.25a | 8.11±2.91a | 12.0±2.51a | 11.8±2.46a | 2.92±0.09a | 3.02±0.24a |
|  |  | Pb500 | 22.3±1.99b | 15.6±1.48ab | 1.74±0.05b | 1.10±0.13b | 2.29±0.10a | 1.84±0.09a | 101±15.6a | 91.6±19.1a | 17.0±3.80a | 9.41±2.59a | 12.4±1.78a | 13.8±2.73a | 2.79±0.21a | 2.88±0.26a |
|  |  | Pb1500 | 18.9±1.48c | 13.4±1.12cd | 1.41±0.04c | 0.86±0.10c | 2.01±0.15b | 1.62±0.11b | 94.4±27.8a | 85.5±13.1a | 16.6±4.01a | 8.42±2.10a | 11.7±2.57a | 13.4±2.86a | 2.50±0.16b | 2.73±0.15ab |
| R+M | -AMF | Pb0 | 21.1±1.06b | 15.5±0.99a | 1.39±0.06c | 0.81±0.04c | 2.20±0.11bc | 1.85±0.15a | 90.1±17.6a | 85.5±15.1a | 16.3±2.98a | 9.72±2.40a | 11.3±2.08a | 11.8±2.15a | 2.24±0.08c | 2.71±0.29ab |
|  |  | Pb500 | 17.5±1.68c | 12.7±0.55b | 1.18±0.05d | 0.70±0.05d | 2.17±0.07bc | 1.63±0.08b | 99.0±12.5a | 96.0±22.5a | 16.5±4.04a | 9.63±2.82a | 11.8±2.49a | 12.4±2.69a | 1.93±0.18d | 2.53±0.16bc |
|  |  | Pb1500 | 13.8±1.19d | 10.3±0.43c | 0.85±0.04e | 0.53±0.03e | 1.76±0.07d | 1.41±0.15c | 100±15.9a | 93.6±22.1a | 16.8±4.03a | 8.52±3.11a | 12.1±2.61a | 12.8±2.44a | 1.93±0.18d | 2.20±0.12c |
|  | +AMF | Pb0 | 24.8±1.03a | 16.3±1.02a | 2.04±0.14a | 1.18±0.08a | 2.30±0.14ab | 2.00±0.05a | 98.4±16.6a | 84.9±15.9a | 16.5±3.23a | 8.44±2.14a | 11.8±3.77a | 13.0±3.58a | 3.02±0.23a | 3.05±0.23a |
|  |  | Pb500 | 21.8±2.13b | 14.8±1.30a | 1.76±0.04b | 1.03±0.08b | 2.43±0.08a | 1.96±0.10a | 91.3±18.3a | 81.3±15.0a | 16.0±1.38a | 7.49±2.38a | 12.1±1.98a | 13.6±3.46a | 2.72±0.24b | 2.73±0.37ab |
|  |  | Pb1500 | 17.9±0.98c | 13.2±1.31b | 1.39±0.05c | 0.81±0.07c | 2.09±0.14c | 1.63±0.09b | 96.5±17.8a | 89.8±13.7a | 15.8±3.28a | 8.07±3.21a | 13.1±2.61a | 13.1±3.50a | 2.38±0.15c | 2.82±0.27ab |
| R+L | -AMF | Pb0 | 16.7±1.24b | 13.0±1.62ab | 0.81±0.07c | 0.80±0.04a | 1.98±0.08abc | 1.43±0.21bc | 96.6±12.8a | 82.7±14.8a | 16.1±2.69a | 7.52±2.54a | 10.4±2.57a | 11.3±2.45a | 2.20±0.19bc | 2.23±0.15ab |
|  |  | Pb500 | 13.5±1.22c | 10.9±0.87bc | 0.74±0.05c | 0.58±0.05c | 1.90±0.13bc | 1.36±0.15c | 90.9±15.0a | 87.8±15.2a | 16.0±4.47a | 8.30±2.98a | 9.80±3.24a | 10.5±2.78a | 1.98±0.20c | 2.18±0.19b |
|  |  | Pb1500 | 13.0±1.38c | 10.6±0.73c | 0.60±0.03d | 0.58±0.06c | 1.79±0.07c | 1.24±0.09c | 95.9±21.5a | 84.0±22.3a | 15.1±3.17a | 8.30±2.61a | 11.7±2.91a | 10.8±2.83a | 1.89±0.15c | 1.89±0.08c |
|  | +AMF | Pb0 | 19.0±1.32a | 13.4±1.74a | 1.54±0.10a | 0.78±0.04ab | 2.09±0.20ab | 1.69±0.12a | 88.0±18.3a | 81.2±12.7a | 15.0±2.50a | 7.59±3.41a | 11.4±3.00a | 12.0±2.14a | 2.52±0.19ab | 2.43±0.10a |
|  |  | Pb500 | 17.7±1.31ab | 12.3±0.77abc | 1.46±0.06b | 0.72±0.08ab | 2.13±0.12a | 1.77±0.15a | 85.8±13.2a | 86.7±23.0a | 15.6±3.80a | 7.65±2.21a | 12.0±2.75a | 11.0±2.19a | 2.62±0.26a | 2.44±0.16a |
|  |  | Pb1500 | 17.0±1.02ab | 12.0±0.87abc | 1.37±0.06c | 0.70±0.06b | 1.96±0.10abc | 1.62±0.08ab | 92.0±17.0a | 90.0±22.5a | 15.3±3.89a | 7.89±2.30a | 12.6±3.67a | 12.1±3.23a | 2.42±0.28b | 2.19±0.18b |

**Table S5.** Macronutrient concentrations in the shoots and roots of *T. pretense*, *M. sativa* and *L. perenne* in different treatments. Values are means ± SD of four replicates. Monocultures of *R. pseudoacacia*, *T. pretense*, *M. sativa* and *L. perenne* are represented by R, T, M and L, respectively. Co-culture planting pattern is represented by the two co-culture species connected with the plus sign (+). The results are reported as the mean (n = 4) ± SD. Different letters indicate that significant differences were detected in macronutrient concentrations of each plant species grown in one planting pattern but at different Pb levels and AMF inoculation status by Duncan's multiple-range tests (*P* < 0.05).

| Plant  species | Planting  pattern | AMF | Pb  level | Macronutrient concentration | | | | | | | |
| --- | --- | --- | --- | --- | --- | --- | --- | --- | --- | --- | --- |
|  |  |  |  | N (g kg^-1^) | | P (g kg^-1^) | | S (g kg^-1^) | | Mg (g kg^-1^) | |
|  |  |  |  | Shoot | Root | Shoot | Root | Shoot | Root | Shoot | Root |
| T | T | -AMF | Pb0 | 25.8±1.88ab | 21.5±2.19a | 1.56±0.09ab | 1.43±0.15ab | 1.67±0.15ab | 1.46±0.12abc | 2.43±0.30ab | 2.63±0.28ab |
|  |  |  | Pb500 | 22.3±1.50bcd | 21.1±3.14a | 1.38±0.14b | 1.10±0.15c | 1.60±0.10bc | 1.35±0.14c | 2.21±0.21b | 2.43±0.21bc |
|  |  |  | Pb1500 | 20.2±1.66d | 18.7±2.86a | 1.13±0.17c | 0.89±0.13d | 1.44±0.14c | 1.38±0.14bc | 1.84±0.09c | 2.24±0.27c |
|  |  | +AMF | Pb0 | 26.5±4.08a | 21.7±2.27a | 1.57±0.15ab | 1.53±0.08a | 1.83±0.06a | 1.60±0.11a | 2.42±0.14ab | 2.46±0.24bc |
|  |  |  | Pb500 | 24.3±2.64abc | 20.1±3.86a | 1.66±0.10a | 1.43±0.12ab | 1.86±0.14a | 1.64±0.07a | 2.69±0.25a | 2.89±0.23a |
|  |  |  | Pb1500 | 20.9±2.32cd | 17.9±3.18a | 1.39±0.08b | 1.27±0.10bc | 1.69±0.13ab | 1.56±0.12ab | 2.28±0.21b | 2.47±0.17bc |
|  | R+T | -AMF | Pb0 | 27.4±2.56a | 23.1±2.26a | 1.59±0.23a | 1.37±0.12a | 1.62±0.10c | 1.45±0.13c | 2.55±0.21a | 2.59±0.29ab |
|  |  |  | Pb500 | 24.6±1.59a | 18.9±3.68ab | 1.42±0.15a | 1.14±0.10b | 1.54±0.12cd | 1.43±0.10c | 2.37±0.29ab | 2.32±0.34b |
|  |  |  | Pb1500 | 19.1±2.29b | 16.7±3.06b | 1.12±0.10b | 0.97±0.14b | 1.44±0.09d | 1.37±0.06c | 2.13±0.21b | 2.28±0.21b |
|  |  | +AMF | Pb0 | 28.2±2.95a | 21.5±4.74ab | 1.56±0.13a | 1.44±0.08a | 1.83±0.10ab | 1.64±0.09b | 2.60±0.17a | 2.84±0.17a |
|  |  |  | Pb500 | 24.4±3.22a | 18.2±2.85ab | 1.66±0.15a | 1.43±0.13a | 1.89±0.12a | 1.79±0.09a | 2.69±0.29a | 2.87±0.10a |
|  |  |  | Pb1500 | 19.9±2.47b | 18.1±3.66ab | 1.44±0.12a | 1.32±0.11a | 1.70±0.11bc | 1.64±0.09b | 2.46±0.19ab | 2.74±0.27a |
| M | M | -AMF | Pb0 | 30.0±2.62a | 20.8±2.10a | 1.54±0.17ab | 1.37±0.10a | 1.47±0.08ab | 1.20±0.08c | 2.49±0.34a | 2.61±0.41ab |
|  |  |  | Pb500 | 20.4±2.19bc | 19.1±2.18a | 1.37±0.14b | 1.10±0.19bc | 1.30±0.05c | 1.25±0.11bc | 2.20±0.15ab | 2.47±0.25ab |
|  |  |  | Pb1500 | 19.0±2.03c | 17.4±2.40a | 1.08±0.14c | 0.94±0.11c | 1.28±0.09c | 1.12±0.09c | 1.95±0.30b | 2.28±0.28b |
|  |  | +AMF | Pb0 | 24.1±3.30ab | 20.8±2.14a | 1.51±0.08ab | 1.50±0.23a | 1.61±0.14a | 1.43±0.12a | 2.45±0.19a | 2.55±0.24ab |
|  |  |  | Pb500 | 21.9±3.52abc | 19.6±1.55a | 1.72±0.13a | 1.47±0.10a | 1.49±0.11ab | 1.39±0.11ab | 2.61±0.30a | 2.79±0.25a |
|  |  |  | Pb1500 | 18.7±1.35c | 17.7±2.23a | 1.42±0.15b | 1.30±0.14ab | 1.42±0.09bc | 1.26±0.06bc | 2.33±0.31ab | 2.55±0.25ab |
|  | R+M | -AMF | Pb0 | 25.3±3.34a | 19.9±2.50a | 1.53±0.13bc | 1.30±0.16bc | 1.41±0.12b | 1.26±0.05b | 2.56±0.26a | 2.51±0.35ab |
|  |  |  | Pb500 | 22.6±1.37ab | 19.0±2.21a | 1.32±0.20cd | 1.18±0.12c | 1.42±0.07b | 1.31±0.11b | 2.41±0.32ab | 2.58±0.22ab |
|  |  |  | Pb1500 | 19.1±1.96b | 18.4±2.50a | 1.16±0.21d | 0.94±0.14d | 1.32±0.12b | 1.22±0.08b | 2.09±0.12b | 2.32±0.13b |
|  |  | +AMF | Pb0 | 23.2±4.18ab | 19.9±1.74a | 1.61±0.15ab | 1.41±0.12ab | 1.61±0.11a | 1.52±0.11a | 2.74±0.28a | 2.43±0.06ab |
|  |  |  | Pb500 | 20.9±3.35ab | 17.7±2.43a | 1.79±0.11a | 1.56±0.08a | 1.75±0.10a | 1.56±0.09a | 2.59±0.09a | 2.75±0.31a |
|  |  |  | Pb1500 | 19.2±1.86b | 18.0±3.35a | 1.48±0.07bc | 1.36±0.09bc | 1.69±0.09a | 1.51±0.12a | 2.71±0.26a | 2.62±0.31ab |
| L | L | -AMF | Pb0 | 16.0±2.27a | 12.8±2.32a | 1.10±0.08b | 0.98±0.13b | 2.01±0.08a | 1.77±0.38a | 1.94±0.13a | 2.04±0.18ab |
|  |  |  | Pb500 | 13.1±2.20bc | 11.5±1.44a | 1.11±0.11b | 0.94±0.13b | 1.78±0.32a | 1.80±0.37a | 1.91±0.19a | 1.87±0.16b |
|  |  |  | Pb1500 | 10.4±1.89c | 9.71±1.58a | 1.15±0.12ab | 1.04±0.06b | 1.87±0.39a | 1.90±0.33a | 1.95±0.24a | 2.06±0.21ab |
|  |  | +AMF | Pb0 | 17.1±1.71a | 12.3±2.36a | 1.32±0.12a | 1.09±0.14ab | 1.57±0.30a | 1.96±0.23a | 1.97±0.22a | 2.10±0.16ab |
|  |  |  | Pb500 | 14.5±1.25ab | 12.2±2.06a | 1.31±0.12a | 1.21±0.05a | 1.88±0.38a | 1.84±0.32a | 2.06±0.26a | 2.21±0.15a |
|  |  |  | Pb1500 | 11.9±1.11bc | 11.0±1.61a | 1.30±0.08a | 1.23±0.08a | 1.88±0.47a | 1.90±0.32a | 2.00±0.21a | 2.05±0.20ab |
|  | R+L | -AMF | Pb0 | 19.1±2.53a | 16.6±2.28a | 1.32±0.10a | 1.14±0.15b | 1.67±0.37a | 1.79±0.58ab | 1.96±0.24a | 2.07±0.25ab |
|  |  |  | Pb500 | 18.4±2.43a | 15.2±1.58a | 1.30±0.07a | 1.18±0.08b | 1.74±0.40a | 1.35±0.06b | 2.02±0.23a | 1.93±0.24b |
|  |  |  | Pb1500 | 17.3±1.60a | 14.0±1.29a | 1.31±0.12a | 1.18±0.10b | 1.74±0.44a | 2.03±0.20a | 2.02±0.13a | 2.12±0.24ab |
|  |  | +AMF | Pb0 | 21.1±3.01a | 17.2±2.46a | 1.48±0.16a | 1.37±0.12a | 1.61±0.45a | 1.74±0.39ab | 1.99±0.27a | 2.23±0.16ab |
|  |  |  | Pb500 | 18.9±2.48a | 14.6±1.42a | 1.48±0.11a | 1.31±0.11ab | 1.83±0.38a | 1.77±0.49ab | 2.10±0.18a | 2.31±0.14a |
|  |  |  | Pb1500 | 19.3±2.52a | 15.2±2.55a | 1.49±0.15a | 1.40±0.10a | 1.94±0.47a | 1.94±0.44ab | 2.12±0.25a | 2.21±0.12ab |

**Table S6.** Multiple ANOVA comparisons of macronutrient concentrations in the shoots of *R. pseudoacacia* affected by neighbor plant (neighbor), AMF inoculation (AMF), Pb level (Pb) and their interactions in different treatments. Monocultures of *R. pseudoacacia*, *T. pretense*, *M. sativa* and *L. perenne* are represented by R, T, M and L, respectively. Co-culture planting pattern is represented by the two co-culture species connected with the plus sign (+). (** *P* < 0.01; * *P* < 0.05; ns, no significance).

| Planting  pattern | Significance | Macronutrient | | | | | | | | | | | | | |
| --- | --- | --- | --- | --- | --- | --- | --- | --- | --- | --- | --- | --- | --- | --- | --- |
|  |  | N | | P | | S | | Na | | K | | Ca | | Mg | |
|  |  | *F* | *P* | *F* | *P* | *F* | *P* | *F* | *P* | *F* | *P* | *F* | *P* | *F* | *P* |
| Mono | AMF | 36.2 | 0.00** | 152 | 0.00** | 24.1 | 0.00** | 0.10 | 0.76ns | 0.20 | 0.66ns | 0.24 | 0.63ns | 48.5 | 0.00** |
|  | Pb | 37.6 | 0.00** | 54.2 | 0.00** | 49.6 | 0.00** | 0.30 | 0.75ns | 0.02 | 0.98ns | 0.94 | 0.41ns | 7.28 | 0.005** |
|  | AMF × Pb | 0.90 | 0.43ns | 0.05 | 0.95ns | 3.84 | 0.04* | 0.02 | 0.99ns | 0.41 | 0.67ns | 0.29 | 0.75ns | 0.73 | 0.50ns |
| R+T | AMF | 40.0 | 0.00** | 397 | 0.00** | 12.4 | 0.00** | 0.22 | 0.64ns | 0.02 | 0.88ns | 0.16 | 0.70ns | 167 | 0.00** |
|  | Pb | 44.8 | 0.00** | 108 | 0.00** | 24.1 | 0.00** | 0.05 | 0.95ns | 0.27 | 0.77ns | 0.25 | 0.79ns | 9.51 | 0.00** |
|  | AMF × Pb | 0.86 | 0.44ns | 4.70 | 0.02* | 3.03 | 0.07ns | 0.30 | 0.75ns | 0.32 | 0.73ns | 0.15 | 0.86ns | 3.07 | 0.07ns |
| Neighbor | | 35.0 | 0.00** | 22.9 | 0.00** | 2.47 | 0.13ns | 0.11 | 0.74ns | 0.00 | 1.00ns | 0.17 | 0.68ns | 11.2 | 0.00** |
| AMF | | 76.0 | 0.00** | 449 | 0.00** | 35.3 | 0.00** | 0.31 | 0.58ns | 0.05 | 0.82ns | 0.39 | 0.54ns | 172 | 0.00** |
| Pb | | 81.7 | 0.00** | 140 | 0.00** | 70.6 | 0.00** | 0.16 | 0.85ns | 0.15 | 0.86ns | 0.50 | 0.61ns | 15.9 | 0.00** |
| Neighbor × AMF | | 0.00 | 0.96ns | 0.23 | 0.63ns | 0.78 | 0.38ns | 0.02 | 0.90ns | 0.18 | 0.67ns | 0.00 | 0.96ns | 2.79 | 0.10ns |
| Neighbor × Pb | | 0.14 | 0.87ns | 0.44 | 0.65ns | 1.87 | 0.17ns | 0.17 | 0.85ns | 0.11 | 0.90ns | 0.65 | 0.53ns | 0.35 | 0.71ns |
| AMF × Pb | | 1.76 | 0.19ns | 1.47 | 0.24ns | 6.62 | 0.00** | 0.10 | 0.90ns | 0.61 | 0.55ns | 0.42 | 0.66ns | 0.99 | 0.38ns |
| Neighbor × AMF × Pb | | 0.00 | 1.00ns | 1.38 | 0.26ns | 0.25 | 0.78ns | 0.23 | 0.80ns | 0.13 | 0.88ns | 0.01 | 0.99ns | 2.05 | 0.14ns |
|  | |  |  |  |  |  |  |  |  |  |  |  |  |  |  |
| R+M | AMF | 48.8 | 0.00** | 398 | 0.00** | 27.8 | 0.00** | 0.03 | 0.88ns | 0.08 | 0.78ns | 1.14 | 0.30ns | 77.7 | 0.00** |
|  | Pb | 51.5 | 0.00** | 139 | 0.00** | 29.8 | 0.00** | 0.14 | 0.87ns | 0.01 | 1.00ns | 0.47 | 0.63ns | 13.3 | 0.00** |
|  | AMF × Pb | 0.13 | 0.88ns | 1.41 | 0.27ns | 2.35 | 0.12ns | 0.50 | 0.61ns | 0.07 | 0.93ns | 0.11 | 0.90ns | 2.20 | 0.14ns |
| Neighbor | | 18.3 | 0.00** | 26.5 | 0.00** | 11.0 | 0.00** | 0.11 | 0.74ns | 0.00 | 0.97ns | 0.08 | 0.78ns | 11.1 | 0.00** |
| AMF | | 84.5 | 0.00** | 428 | 0.00** | 51.1 | 0.00** | 0.11 | 0.74ns | 0.03 | 0.87ns | 1.31 | 0.26ns | 123 | 0.00** |
| Pb | | 88.4 | 0.00** | 151 | 0.00** | 74.3 | 0.00** | 0.09 | 0.92ns | 0.00 | 1.00ns | 1.29 | 0.29ns | 18.5 | 0.00** |
| Neighbor × AMF | | 0.37 | 0.55ns | 0.43 | 0.52ns | 0.00 | 0.97ns | 0.01 | 0.91ns | 0.27 | 0.61ns | 0.29 | 0.59ns | 0.48 | 0.49ns |
| Neighbor × Pb | | 0.50 | 0.61ns | 0.61 | 0.55ns | 5.25 | 0.01** | 0.36 | 0.70ns | 0.02 | 0.98ns | 0.00 | 1.00ns | 1.59 | 0.22ns |
| AMF × Pb | | 0.81 | 0.46ns | 0.48 | 0.63ns | 5.48 | 0.01** | 0.28 | 0.76ns | 0.37 | 0.69ns | 0.16 | 0.85ns | 0.14 | 0.87ns |
| Neighbor × AMF × Pb | | 0.24 | 0.79ns | 0.32 | 0.73ns | 0.67 | 0.52ns | 0.21 | 0.81ns | 0.18 | 0.83ns | 0.20 | 0.82ns | 2.63 | 0.09ns |
|  | |  |  |  |  |  |  |  |  |  |  |  |  |  |  |
| R+L | AMF | 46.1 | 0.00** | 767 | 0.00** | 11.5 | 0.00** | 0.75 | 0.40ns | 0.08 | 0.78ns | 1.14 | 0.30ns | 31.7 | 0.00** |
|  | Pb | 10.9 | 0.00** | 17.0 | 0.00** | 3.85 | 0.04* | 0.24 | 0.79ns | 0.06 | 0.94ns | 0.47 | 0.63ns | 1.91 | 0.18ns |
|  | AMF × Pb | 1.30 | 0.30ns | 0.57 | 0.58ns | 0.53 | 0.60ns | 0.04 | 0.96ns | 0.07 | 0.94ns | 0.11 | 0.90ns | 1.15 | 0.34ns |
| Neighbor | | 17.0 | 0.00** | 46.3 | 0.00** | 4.91 | 0.03* | 1.50 | 0.23ns | 0.58 | 0.45ns | 0.08 | 0.78ns | 2.20 | 0.15ns |
| AMF | | 81.1 | 0.00** | 569 | 0.00** | 33.7 | 0.00** | 0.67 | 0.42ns | 0.02 | 0.88ns | 1.31 | 0.26ns | 79.5 | 0.00** |
| Pb | | 45.0 | 0.00** | 71.0 | 0.00** | 38.1 | 0.00** | 0.01 | 0.99ns | 0.05 | 0.95ns | 1.29 | 0.29ns | 8.22 | 0.00** |
| Neighbor × AMF | | 0.00 | 0.97ns | 4.03 | 0.05ns | 0.72 | 0.40ns | 0.14 | 0.72ns | 0.27 | 0.61ns | 0.29 | 0.59ns | 0.62 | 0.44ns |
| Neighbor × Pb | | 6.94 | 0.00** | 21.2 | 0.00** | 11.3 | 0.00** | 0.53 | 0.59ns | 0.02 | 0.98ns | 0.00 | 1.00ns | 0.89 | 0.42ns |
| AMF × Pb | | 2.12 | 0.14ns | 0.03 | 0.98ns | 3.31 | 0.05* | 0.03 | 0.97ns | 0.17 | 0.85ns | 0.16 | 0.85ns | 0.97 | 0.39ns |
| Neighbor × AMF × Pb | | 0.04 | 0.96ns | 0.31 | 0.74ns | 0.70 | 0.51ns | 0.03 | 0.97ns | 0.36 | 0.70ns | 0.20 | 0.82ns | 0.98 | 0.39ns |

**Table S7.** Multiple ANOVA comparisons of macronutrient concentrations in the roots of *R. pseudoacacia* affected by neighbor plant (neighbor), AMF inoculation (AMF), Pb level (Pb) and their interactions in different treatments. Monocultures of *R. pseudoacacia*, *T. pretense*, *M. sativa* and *L. perenne* are represented by R, T, M and L, respectively. Co-culture planting pattern is represented by the two co-culture species connected with the plus sign (+). (** *P* < 0.01; * *P* < 0.05; ns, no significance).

| Planting  pattern | Significance | Macronutrient | | | | | | | | | | | | | |
| --- | --- | --- | --- | --- | --- | --- | --- | --- | --- | --- | --- | --- | --- | --- | --- |
|  |  | N | | P | | S | | Na | | K | | Ca | | Mg | |
|  |  | *F* | *P* | *F* | *P* | *F* | *P* | *F* | *P* | *F* | *P* | *F* | *P* | *F* | *P* |
| Mono | AMF | 13.6 | 0.00** | 117 | 0.00** | 4.46 | 0.049* | 0.16 | 0.69ns | 0.90 | 0.36ns | 0.10 | 0.76ns | 15.5 | 0.00** |
|  | Pb | 22.0 | 0.00** | 32.0 | 0.00** | 62.9 | 0.00** | 0.38 | 0.69ns | 0.15 | 0.86ns | 0.23 | 0.80ns | 18.5 | 0.00** |
|  | AMF × Pb | 0.87 | 0.44ns | 0.32 | 0.73ns | 5.68 | 0.012* | 0.16 | 0.85ns | 0.14 | 0.87ns | 0.02 | 0.98ns | 0.10 | 0.90ns |
| R+T | AMF | 15.6 | 0.00** | 185 | 0.00** | 24.7 | 0.00** | 0.02 | 0.90ns | 0.79 | 0.39ns | 0.48 | 0.50ns | 33.4 | 0.00** |
|  | Pb | 13.8 | 0.00** | 48.4 | 0.00** | 21.9 | 0.00** | 0.13 | 0.88ns | 0.10 | 0.91ns | 0.11 | 0.90ns | 4.89 | 0.02* |
|  | AMF × Pb | 0.35 | 0.71ns | 5.35 | 0.02* | 0.99 | 0.39ns | 0.34 | 0.72ns | 0.28 | 0.76ns | 0.01 | 1.00ns | 0.45 | 0.65ns |
| Neighbor | | 19.7 | 0.00** | 15.3 | 0.00** | 7.23 | 0.01* | 0.00 | 0.97ns | 1.00 | 0.32ns | 0.50 | 0.48ns | 6.98 | 0.01* |
| AMF | | 29.1 | 0.00** | 301 | 0.00** | 26.0 | 0.00** | 0.14 | 0.71ns | 1.69 | 0.20ns | 0.54 | 0.47ns | 49.1 | 0.00** |
| Pb | | 34.0 | 0.00** | 80.1 | 0.00** | 74.7 | 0.00** | 0.37 | 0.70ns | 0.15 | 0.86ns | 0.25 | 0.78ns | 18.6 | 0.00** |
| Neighbor × AMF | | 0.36 | 0.55ns | 7.86 | 0.01** | 5.88 | 0.02* | 0.04 | 0.85ns | 0.00 | 0.97ns | 0.11 | 0.74ns | 5.73 | 0.02* |
| Neighbor × Pb | | 0.59 | 0.56ns | 1.90 | 0.17ns | 2.50 | 0.10ns | 0.15 | 0.86ns | 0.10 | 0.91ns | 0.07 | 0.94ns | 0.62 | 0.54ns |
| AMF × Pb | | 0.11 | 0.89ns | 2.47 | 0.10ns | 4.52 | 0.02ns | 0.08 | 0.93ns | 0.41 | 0.67ns | 0.02 | 0.98ns | 0.40 | 0.67ns |
| Neighbor × AMF × Pb | | 1.03 | 0.37ns | 3.70 | 0.04* | 1.26 | 0.30ns | 0.42 | 0.66ns | 0.01 | 0.99ns | 0.00 | 1.00ns | 0.26 | 0.77ns |
|  | |  |  |  |  |  |  |  |  |  |  |  |  |  |  |
| R+M | AMF | 23.1 | 0.00** | 171 | 0.00** | 28.0 | 0.00** | 0.86 | 0.37ns | 1.36 | 0.26ns | 0.56 | 0.47ns | 13.6 | 0.00** |
|  | Pb | 35.0 | 0.00** | 56.7 | 0.00** | 28.9 | 0.00** | 0.27 | 0.77ns | 0.17 | 0.84ns | 0.10 | 0.91ns | 4.31 | 0.03* |
|  | AMF × Pb | 2.21 | 0.14ns | 1.14 | 0.34ns | 1.27 | 0.31ns | 0.40 | 0.68ns | 0.20 | 0.82ns | 0.05 | 0.95ns | 1.33 | 0.29ns |
| Neighbor | | 11.2 | 0.00** | 4.11 | 0.05* | 22.2 | 0.00** | 0.03 | 0.86ns | 2.57 | 0.12ns | 0.57 | 0.45ns | 15.1 | 0.00** |
| AMF | | 35.3 | 0.00** | 280 | 0.00** | 28.3 | 0.00** | 0.88 | 0.36ns | 2.25 | 0.14ns | 0.61 | 0.44ns | 28.0 | 0.00** |
| Pb | | 55.4 | 0.00** | 84.5 | 0.00** | 85.7 | 0.00** | 0.03 | 0.98ns | 0.21 | 0.82ns | 0.29 | 0.75ns | 17.0 | 0.00** |
| Neighbor × AMF | | 0.19 | 0.67ns | 0.09 | 0.77ns | 6.33 | 0.02* | 0.14 | 0.71ns | 0.03 | 0.86ns | 0.16 | 0.70ns | 0.72 | 0.40ns |
| Neighbor × Pb | | 0.17 | 0.85ns | 0.44 | 0.65ns | 2.09 | 0.14ns | 0.63 | 0.54ns | 0.12 | 0.89ns | 0.00 | 1.00ns | 0.84 | 0.44ns |
| AMF × Pb | | 2.85 | 0.07ns | 0.60 | 0.55ns | 5.11 | 0.01* | 0.51 | 0.61ns | 0.02 | 0.98ns | 0.01 | 0.99ns | 1.00 | 0.38ns |
| Neighbor × AMF × Pb | | 0.09 | 0.91ns | 0.73 | 0.49ns | 1.37 | 0.27ns | 0.03 | 0.86ns | 0.32 | 0.73ns | 0.07 | 0.93ns | 0.85 | 0.44ns |
|  | |  |  |  |  |  |  |  |  |  |  |  |  |  |  |
| R+L | AMF | 3.75 | 0.07ns | 9.82 | 0.006** | 38.7 | 0.00** | 0.02 | 0.88ns | 0.04 | 0.85ns | 0.80 | 0.38ns | 17.8 | 0.00** |
|  | Pb | 4.52 | 0.03* | 16.8 | 0.00** | 2.35 | 0.12ns | 0.20 | 0.82ns | 0.04 | 0.97ns | 0.30 | 0.75ns | 9.52 | 0.00** |
|  | AMF × Pb | 0.36 | 0.70ns | 4.70 | 0.02* | 0.67 | 0.52ns | 0.10 | 0.91ns | 0.09 | 0.92ns | 0.09 | 0.92ns | 0.25 | 0.78ns |
| Neighbor | | 4.27 | 0.05* | 33.1 | 0.00** | 6.39 | 0.02* | 0.54 | 0.47ns | 6.37 | 0.02ns | 7.93 | 0.01ns | 18.3 | 0.00** |
| AMF | | 14.8 | 0.00** | 108 | 0.00** | 39.3 | 0.00** | 0.03 | 0.87ns | 0.63 | 0.43ns | 0.77 | 0.39ns | 32.8 | 0.00** |
| Pb | | 21.1 | 0.00** | 45.8 | 0.00** | 32.4 | 0.00** | 0.13 | 0.88ns | 0.07 | 0.93ns | 0.10 | 0.90ns | 27.0 | 0.00** |
| Neighbor × AMF | | 0.78 | 0.38ns | 41.7 | 0.00** | 14.8 | 0.00** | 0.15 | 0.70ns | 0.28 | 0.60ns | 0.21 | 0.65nsns | 0.07 | 0.79ns |
| Neighbor × Pb | | 2.43 | 0.10ns | 6.17 | 0.01** | 12.5 | 0.00** | 0.44 | 0.65ns | 0.11 | 0.90ns | 0.43 | 0.65ns | 2.65 | 0.08ns |
| AMF × Pb | | 1.06 | 0.36ns | 2.98 | 0.06ns | 4.08 | 0.03* | 0.10 | 0.90ns | 0.05 | 0.95ns | 0.09 | 0.91ns | 0.30 | 0.74ns |
| Neighbor × AMF × Pb | | 0.08 | 0.92ns | 1.12 | 0.34ns | 0.61 | 0.55ns | 0.15 | 0.86ns | 0.18 | 0.84ns | 0.03 | 0.97ns | 0.02 | 0.98ns |

**Table S8.** Multiple ANOVA comparisons of macronutrient concentrations in the shoots and roots of *T. pretense*, *M. sativa* and *L. perenne* affected by neighbor plant (neighbor), AMF inoculation (AMF), Pb level (Pb) and their interactions in different treatments. Monocultures of *R. pseudoacacia*, *T. pretense*, *M. sativa* and *L. perenne* are represented by R, T, M and L, respectively. Co-culture planting pattern is represented by the two co-culture species connected with the plus sign (+). (** *P* < 0.01; * *P* < 0.05; ns, no significance).

| Plant  species | Planting  pattern | significance | Macronutrient in shoot | | | | | | | | Macronutrient in root | | | | | | | |
| --- | --- | --- | --- | --- | --- | --- | --- | --- | --- | --- | --- | --- | --- | --- | --- | --- | --- | --- |
|  |  |  | N | | P | | S | | Mg | | N | | P | | S | | Mg | |
|  |  |  | *F* | *P* | *F* | *P* | *F* | *P* | *F* | *P* | *F* | *P* | *F* | *P* | *F* | *P* | *F* | *P* |
| T | T | AMF | 1.21 | 0.29ns | 12.6 | 0.00** | 19.6 | 0.00** | 12.3 | 0.00** | 0.23 | 0.64ns | 27.9 | 0.00** | 17.4 | 0.00** | 3.24 | 0.09ns |
|  |  | Pb | 9.97 | 0.00** | 13.9 | 0.00** | 5.27 | 0.02* | 8.53 | 0.00** | 2.55 | 0.11ns | 20.8 | 0.00** | 0.51 | 0.61ns | 3.47 | 0.053ns |
|  |  | AMF × Pb | 0.18 | 0.84ns | 3.05 | 0.07ns | 0.40 | 0.68ns | 3.22 | 0.06ns | 0.09 | 0.92ns | 3.10 | 0.07ns | 0.83 | 0.45ns | 3.66 | 0.046* |
|  | R+T | AMF | 0.17 | 0.69ns | 8.30 | 0.01** | 37.8 | 0.00** | 5.90 | 0.03* | 0.05 | 0.82ns | 25.9 | 0.00** | 49.5 | 0.00** | 18.1 | 0.00** |
|  |  | Pb | 21.4 | 0.00** | 9.15 | 0.00** | 5.32 | 0.02* | 3.40 | 0.06ns | 4.44 | 0.03* | 10.1 | 0.00** | 2.36 | 0.12ns | 1.55 | 0.24ns |
|  |  | AMF × Pb | 0.09 | 0.91ns | 2.93 | 0.08ns | 0.79 | 0.47ns | 0.96 | 0.40ns | 0.40 | 0.68ns | 3.39 | 0.06ns | 1.60 | 0.23ns | 0.82 | 0.46ns |
|  | Neighbor | | 0.77 | 0.39ns | 0.25 | 0.62ns | 0.05 | 0.83ns | 5.86 | 0.02* | 0.64 | 0.43ns | 0.02 | 0.89ns | 2.76 | 0.11ns | 1.53 | 0.22ns |
|  | AMF | | 1.12 | 0.30ns | 20.1 | 0.00** | 54.6 | 0.00** | 17.3 | 0.00** | 0.23 | 0.63ns | 53.8 | 0.00** | 58.7 | 0.00** | 18.5 | 0.00** |
|  | Pb | | 30.3 | 0.00** | 22.2 | 0.00** | 10.5 | 0.00** | 11.0 | 0.00** | 6.51 | 0.00** | 30.3 | 0.00** | 1.55 | 0.23ns | 3.73 | 0.03* |
|  | Neighbor × AMF | | 0.23 | 0.64ns | 0.00 | 0.95ns | 0.54 | 0.47ns | 0.31 | 0.58ns | 0.02 | 0.89ns | 0.20 | 0.66ns | 1.37 | 0.25ns | 3.21 | 0.08ns |
|  | Neighbor × Pb | | 1.35 | 0.27ns | 0.01 | 0.99ns | 0.05 | 0.96ns | 0.47 | 0.63ns | 0.76 | 0.47ns | 1.52 | 0.23ns | 0.93 | 0.41ns | 1.25 | 0.30ns |
|  | AMF × Pb | | 0.01 | 0.99ns | 5.81 | 0.01** | 1.00 | 0.38ns | 3.73 | 0.03ns | 0.16 | 0.85ns | 6.46 | 0.00** | 2.24 | 0.12ns | 3.92 | 0.03* |
|  | Neighbor × AMF × Pb | | 0.26 | 0.77ns | 0.15 | 0.86ns | 0.14 | 0.87ns | 0.23 | 0.79ns | 0.37 | 0.69ns | 0.00 | 1.00ns | 0.02 | 0.98ns | 0.48 | 0.62ns |
|  |  | |  |  |  |  |  |  |  |  |  |  |  |  |  |  |  |  |
| M | M | AMF | 0.03 | 0.86ns | 15.3 | 0.00** | 15.8 | 0.00** | 4.82 | 0.04* | 0.10 | 0.76ns | 21.1 | 0.00** | 18.4 | 0.00** | 2.29 | 0.15ns |
|  |  | Pb | 11.5 | 0.00** | 11.5 | 0.00** | 8.56 | 0.00** | 3.28 | 0.06ns | 4.70 | 0.02* | 8.36 | 0.00** | 4.49 | 0.03* | 1.24 | 0.31ns |
|  |  | AMF × Pb | 0.85 | 0.44ns | 4.80 | 0.02* | 0.12 | 0.89ns | 1.67 | 0.22ns | 0.02 | 0.98ns | 1.56 | 0.24ns | 0.62 | 0.55ns | 1.03 | 0.38ns |
|  | R+M | AMF | 1.21 | 0.29ns | 22.5 | 0.00** | 53.6 | 0.00** | 11.4 | 0.00** | 0.29 | 0.60ns | 37.6 | 0.00** | 42.3 | 0.00* | 1.70 | 0.21ns |
|  |  | Pb | 6.34 | 0.01** | 6.84 | 0.01** | 1.37 | 0.28ns | 2.26 | 0.13ns | 1.13 | 0.35ns | 7.86 | 0.00** | 0.37 | 0.50ns | 1.61 | 0.23ns |
|  |  | AMF × Pb | 0.31 | 0.73ns | 3.29 | 0.06ns | 1.94 | 0.17ns | 2.35 | 0.12ns | 0.16 | 0.86ns | 3.76 | 0.04* | 0.15 | 0.86ns | 1.22 | 0.32ns |
|  | Neighbor | | 0.00 | 0.96ns | 0.95 | 0.34ns | 13.4 | 0.00** | 5.75 | 0.02* | 0.40 | 0.53ns | 0.05 | 0.82ns | 18.3 | 0.00** | 0.01 | 0.93ns |
|  | AMF | | 0.87 | 0.36ns | 37.7 | 0.00** | 63.6 | 0.00** | 15.0 | 0.00** | 0.04 | 0.84ns | 55.1 | 0.00** | 60.2 | 0.00** | 3.99 | 0.05ns |
|  | Pb | | 17.1 | 0.00** | 17.7 | 0.00** | 4.00 | 0.03* | 5.23 | 0.01** | 4.59 | 0.02* | 14.9 | 0.00** | 4.49 | 0.02* | 2.32 | 0.11ns |
|  | Neighbor × AMF | | 0.47 | 0.50ns | 0.77 | 0.39ns | 5.96 | 0.02* | 0.31 | 0.58ns | 0.37 | 0.55ns | 0.07 | 0.79ns | 2.85 | 0.10ns | 0.08 | 0.79ns |
|  | Neighbor × Pb | | 0.29 | 0.75ns | 0.17 | 0.84ns | 6.00 | 0.01** | 0.45 | 0.64ns | 0.64 | 0.53ns | 1.40 | 0.26ns | 1.00 | 0.38ns | 0.48 | 0.62ns |
|  | AMF × Pb | | 0.62 | 0.55ns | 7.67 | 0.00** | 1.03 | 0.37ns | 2.83 | 0.07ns | 0.05 | 0.95ns | 4.75 | 0.02* | 0.22 | 0.81ns | 2.10 | 0.14ns |
|  | Neighbor × AMF × Pb | | 0.50 | 0.61ns | 0.28 | 0.76ns | 0.72 | 0.49ns | 1.10 | 0.35ns | 0.15 | 0.86ns | 0.10 | 0.91ns | 0.48 | 0.62ns | 0.13 | 0.88ns |
|  |  | |  |  |  |  |  |  |  |  |  |  |  |  |  |  |  |  |
| L | L | AMF | 3.25 | 0.09ns | 19.4 | 0.00** | 0.61 | 0.44ns | 0.77 | 0.39ns | 0.38 | 0.54ns | 19.9 | 0.00** | 0.33 | 0.57ns | 3.19 | 0.09ns |
|  |  | Pb | 17.9 | 0.00** | 0.06 | 0.94ns | 0.12 | 0.89ns | 0.05 | 0.96ns | 2.76 | 0.09ns | 1.99 | 0.17ns | 0.13 | 0.88ns | 0.08 | 0.92ns |
|  |  | AMF × Pb | 0.04 | 0.96ns | 0.21 | 0.81ns | 1.35 | 0.28ns | 0.17 | 0.85ns | 0.44 | 0.65ns | 1.23 | 0.32ns | 0.18 | 0.84ns | 2.19 | 0.14ns |
|  | R+L | AMF | 2.21 | 0.15ns | 0.25 | 0.62ns | 0.19 | 0.67ns | 0.63 | 0.44ns | 0.25 | 0.62ns | 17.5 | 0.00** | 0.34 | 0.57ns | 6.61 | 0.02* |
|  |  | Pb | 1.23 | 0.32ns | 3.19 | 0.07ns | 0.50 | 0.62ns | 0.44 | 0.65ns | 3.19 | 0.07ns | 0.33 | 0.73ns | 2.26 | 0.13ns | 0.10 | 0.90ns |
|  |  | AMF × Pb | 0.23 | 0.79ns | 0.43 | 0.66ns | 0.19 | 0.83ns | 0.05 | 0.95ns | 0.43 | 0.66ns | 0.54 | 0.59ns | 1.03 | 0.38ns | 1.14 | 0.34ns |
|  | Neighbor | | 69.2 | 0.00** | 29.1 | 0.00** | 0.47 | 0.50ns | 1.08 | 0.31ns | 46.6 | 0.00** | 33.6 | 0.00** | 0.73 | 0.40ns | 2.67 | 0.11ns |
|  | AMF | | 5.11 | 0.03* | 30.2 | 0.00** | 0.03 | 0.87ns | 1.39 | 0.25ns | 0.63 | 0.43ns | 37.2 | 0.00** | 0.66 | 0.42ns | 9.63 | 0.00** |
|  | Pb | | 11.2 | 0.00** | 0.05 | 0.95ns | 0.57 | 0.57ns | 0.38 | 0.69ns | 5.42 | 0.01** | 1.76 | 0.19ns | 1.92 | 0.16ns | 0.16 | 0.86ns |
|  | Neighbor × AMF | | 0.02 | 0.89ns | 0.07 | 0.79ns | 0.69 | 0.41ns | 0.00 | 0.97ns | 0.01 | 0.95ns | 0.01 | 0.94ns | 0.01 | 0.93ns | 0.49 | 0.49ns |
|  | Neighbor × Pb | | 2.84 | 0.07ns | 0.02 | 0.98ns | 0.12 | 0.89ns | 0.13 | 0.88ns | 0.54 | 0.59ns | 0.41 | 0.67ns | 0.89 | 0.42ns | 0.03 | 0.97ns |
|  | AMF × Pb | | 0.12 | 0.89ns | 0.05 | 0.95ns | 1.08 | 0.35ns | 0.15 | 0.87ns | 0.50 | 0.61ns | 0.15 | 0.86ns | 0.60 | 0.55ns | 3.19 | 0.05ns |
|  | Neighbor × AMF × Pb | | 0.21 | 0.81ns | 0.15 | 0.86ns | 0.24 | 0.79ns | 0.07 | 0.94ns | 0.36 | 0.70ns | 1.55 | 0.23ns | 0.78 | 0.47ns | 0.04 | 0.96ns |

**Table S9.** Pb concentrations in shoots and roots, and TF values of *R. pseudoacacia* in different treatments. Values are means ± SD of four replicates. Monocultures of *R. pseudoacacia*, *T. pretense*, *M. sativa* and *L. perenne* are represented by R, T, M and L, respectively. Co-culture planting pattern is represented by the two co-culture species connected with the plus sign (+). The results are reported as the mean (n = 4) ± SD. Different letters indicate that significant differences were detected in Pb concentrations or TFs of each plant species grown in one planting pattern but at different Pb levels and AMF inoculation status by Duncan's multiple-range tests (*P* < 0.05).

| Plant  species | Planting  pattern | AMF | Pb concentration (mg kg^-1^) | | | | | | | | |
| --- | --- | --- | --- | --- | --- | --- | --- | --- | --- | --- | --- |
|  |  |  | Shoot | | | Root | | | TF | | |
|  |  |  | Pb0 | Pb500 | Pb1500 | Pb0 | Pb500 | Pb1500 | Pb0 | Pb500 | Pb1500 |
| R | R | -AMF | 8.78±1.76c | 201±25.0b | 289±21.3a | 10.1±1.76e | 289±13.8d | 441±25.8b | 0.88±0.17a | 0.70±0.09b | 0.66±0.03b |
|  |  | +AMF | 10.1±2.19c | 211±19.9b | 219±28.9b | 16.7±2.61e | 371±22.2c | 605±22.9a | 0.61±0.15b | 0.57±0.05b | 0.36±0.05c |
|  | R+T | -AMF | 11.3±1.74d | 232±21.9c | 358±24.6a | 14.3±2.43e | 323±20.5d | 491±19.5b | 0.80±0.16a | 0.72±0.10ab | 0.73±0.07ab |
|  |  | +AMF | 14.1±1.37d | 246±22.0bc | 262±24.1b | 16.6±4.17e | 410±30.9c | 661±26.9a | 0.88±0.14a | 0.60±0.07b | 0.40±0.03c |
|  | R+M | -AMF | 10.6±2.28d | 225±18.0c | 353±23.7a | 13.0±0.91e | 330±18.3d | 502±19.3b | 0.82±0.15a | 0.69±0.08ab | 0.71±0.07ab |
|  |  | +AMF | 13.3±1.71d | 251±22.9bc | 269±32.7b | 16.6±2.75e | 423±23.2c | 656±24.5a | 0.82±0.20a | 0.60±0.07b | 0.41±0.06c |
|  | R+L | -AMF | 7.11±1.23c | 181±31.4b | 295±34.8a | 11.4±2.11e | 289±39.2d | 437±29.4b | 0.64±0.16a | 0.64±0.15a | 0.67±0.07a |
|  |  | +AMF | 11.7±2.32c | 213±26.1b | 202±21.7b | 18.1±1.45e | 369±43.5c | 594±20.8a | 0.65±0.14a | 0.58±0.08a | 0.34±0.03b |
| T | T | -AMF | 8.71±1.86c | 233±36.5b | 347±47.2a | 10.4±2.43e | 301±39.6d | 471±21.5b | 0.90±0.32a | 0.77±0.10ab | 0.74±0.08ab |
|  |  | +AMF | 11.8±2.09c | 232±34.5b | 237±23.2b | 12.1±1.96e | 417±23.6c | 669±31.7a | 0.98±0.12a | 0.56±0.08cd | 0.35±0.02d |
|  | R+T | -AMF | 11.9±2.22c | 285±39.6b | 386±38.0a | 12.3±2.06e | 365±41.2d | 523±27.0b | 1.00±0.30a | 0.78±0.10abc | 0.74±0.04bc |
|  |  | +AMF | 8.81±1.62c | 268±32.8b | 257±22.7b | 11.1±2.67e | 486±21.7c | 706±14.5a | 0.82±0.18ab | 0.55±0.09cd | 0.36±0.03d |
| M | M | -AMF | 9.52±1.93c | 221±33.5b | 351±40.8a | 11.0±2.17e | 276±42.6d | 518±39.0b | 0.88±0.16a | 0.82±0.17ab | 0.68±0.04ab |
|  |  | +AMF | 8.56±1.12c | 236±24.7b | 247±24.3b | 12.1±2.10e | 380±42.0c | 627±34.3a | 0.73±0.21ab | 0.63±0.11b | 0.40±0.06c |
|  | R+M | -AMF | 9.82±1.73c | 271±23.3b | 402±79.2a | 11.3±2.83e | 302±22.8d | 555±34.1b | 0.94±0.41a | 0.90±0.06a | 0.73±0.16ab |
|  |  | +AMF | 8.75±1.13c | 262±21.3b | 278±33.7b | 11.2±1.86e | 437±28.9c | 661±33.0a | 0.81±0.23a | 0.60±0.07ab | 0.42±0.06b |
| L | L | -AMF | 7.54±1.71c | 141±21.8b | 254±38.7a | 10.1±2.20d | 190±28.5c | 318±29.7b | 0.77±0.21a | 0.75±0.15a | 0.80±0.12a |
|  |  | +AMF | 7.97±2.55c | 143±34.9b | 158±35.2b | 10.7±3.01d | 293±38.3b | 418±34.8a | 0.79±0.35a | 0.50±0.18ab | 0.38±0.08b |
|  | R+L | -AMF | 9.96±2.06c | 159±29.8b | 291±31.8a | 11.3±2.51d | 237±27.7c | 351±29.4b | 0.95±0.42a | 0.67±0.08ab | 0.84±0.14a |
|  |  | +AMF | 10.5±1.88c | 159±27.5b | 188±24.7b | 12.5±1.98d | 326±24.8b | 452±32.6a | 0.84±0.05a | 0.49±0.07b | 0.42±0.06b |

**Table S10.** Multiple ANOVA comparisons of Pb concentration in the shoots and roots of *R. pseudoacacia* affected by neighbor plant (neighbor), AMF inoculation (AMF), Pb level (Pb) and their interactions in different treatments. Monocultures of *R. pseudoacacia*, *T. pretense*, *M. sativa* and *L. perenne* are represented by R, T, M and L, respectively. Co-culture planting pattern is represented by the two co-culture species connected with the plus sign (+). (** *P* < 0.01; * *P* < 0.05; ns, no significance).

| Planting  pattern | Significance | Pb concentration | | | | | |
| --- | --- | --- | --- | --- | --- | --- | --- |
|  |  | Shoot | | Root | | TF | |
|  |  | *F* | *P* | *F* | *P* | *F* | *P* |
| R | AMF | 5.80 | 0.03* | 136 | 0.00** | 29.6 | 0.00** |
|  | Pb | 349 | 0.00** | 1687 | 0.00** | 10.6 | 0.00** |
|  | AMF × Pb | 10.1 | 0.00** | 39.5 | 0.00** | 1.54 | 0.24ns |
| R+T | AMF | 11.6 | 0.00** | 107 | 0.00** | 8.29 | 0.01** |
|  | Pb | 536 | 0.00** | 1536 | 0.00** | 13.8 | 0.00** |
|  | AMF × Pb | 20.5 | 0.00** | 33.6 | 0.00** | 7.20 | 0.01** |
| Neighbor | | 30.1 | 0.00** | 30.5 | 0.00** | 3.78 | 0.06ns |
| AMF | | 16.8 | 0.00** | 239 | 0.00** | 34.1 | 0.00** |
| Pb | | 871 | 0.00** | 3194 | 0.00** | 24.1 | 0.00** |
| Neighbor × AMF | | 0.39 | 0.53ns | 0.04 | 0.85ns | 2.86 | 0.10ns |
| Neighbor × Pb | | 7.28 | 0.00** | 7.39 | 0.00** | 0.38 | 0.69ns |
| AMF × Pb | | 29.5 | 0.00** | 72.2 | 0.00** | 5.02 | 0.01* |
| Neighbor × AMF × Pb | | 0.73 | 0.49ns | 0.08 | 0.92ns | 3.95 | 0.03* |
|  | |  |  |  |  |  |  |
| R+M | AMF | 4.92 | 0.04* | 136 | 0.00** | 6.79 | 0.02* |
|  | Pb | 469 | 0.00** | 2117 | 0.00** | 10.3 | 0.00** |
|  | AMF × Pb | 16.4 | 0.00** | 37.1 | 0.00** | 3.31 | 0.06ns |
| Neighbor | | 27.6 | 0.00** | 45.8 | 0.00** | 1.86 | 0.18ns |
| AMF | | 10.7 | 0.00** | 272 | 0.00** | 30.7 | 0.00** |
| Pb | | 815 | 0.00** | 3790 | 0.00** | 20.5 | 0.00** |
| Neighbor × AMF | | 0.01 | 0.94ns | 0.01 | 0.94ns | 2.59 | 0.12ns |
| Neighbor × Pb | | 7.30 | 0.00** | 10.9 | 0.00** | 0.36 | 0.70ns |
| AMF × Pb | | 26.2 | 0.00** | 76.3 | 0.00** | 3.30 | 0.048* |
| Neighbor × AMF × Pb | | 0.55 | 0.58ns | 0.35 | 0.71ns | 1.79 | 0.18ns |
|  | |  |  |  |  |  |  |
| R+L | AMF | 3.68 | 0.07ns | 50.1 | 0.00** | 7.19 | 0.02* |
|  | Pb | 227 | 0.00** | 650 | 0.00** | 3.09 | 0.07ns |
|  | AMF × Pb | 15.3 | 0.00** | 14.3 | 0.00** | 5.16 | 0.02* |
| Neighbor | | 0.62 | 0.44ns | 0.12 | 0.73ns | 1.91 | 0.18ns |
| AMF | | 9.08 | 0.01** | 149 | 0.00** | 31.8 | 0.00** |
| Pb | | 553 | 0.00** | 1891 | 0.00** | 12.0 | 0.00** |
| Neighbor × AMF | | 0.00 | 0.95ns | 0.06 | 0.81ns | 2.75 | 0.11ns |
| Neighbor × Pb | | 0.18 | 0.84ns | 0.16 | 0.86ns | 0.94 | 0.40ns |
| AMF × Pb | | 25.4 | 0.00** | 42.9 | 0.00** | 4.81 | 0.014* |
| Neighbor × AMF × Pb | | 1.02 | 0.37ns | 0.03 | 0.97ns | 2.26 | 0.12ns |

**Table S11.** Multiple ANOVA comparisons of Pb concentration in the shoots and roots of *T. pretense*, *M. sativa* and *L. perenne* affected by neighbor plant (neighbor), AMF inoculation (AMF), Pb level (Pb) and their interactions in different treatments. Monocultures of *R. pseudoacacia*, *T. pretense*, *M. sativa* and *L. perenne* are represented by R, T, M and L, respectively. Co-culture planting pattern is represented by the two co-culture species connected with the plus sign (+). (** *P* < 0.01; * *P* < 0.05; ns, no significance).

| Species | Planting  pattern | Significance | Pb concentration | | | | | |
| --- | --- | --- | --- | --- | --- | --- | --- | --- |
|  |  |  | Shoot | | Root | | TF | |
|  |  |  | *F* | *P* | *F* | *P* | *F* | *P* |
| T | T | AMF | 8.88 | 0.01** | 108 | 0.00** | 7.47 | 0.01* |
|  |  | Pb | 200 | 0.00** | 1062 | 0.00** | 13.5 | 0.00** |
|  |  | AMF × Pb | 9.31 | 0.00** | 32.1 | 0.00** | 4.49 | 0.03* |
|  | R+T | AMF | 19.2 | 0.00** | 117 | 0.00** | 17.2 | 0.00** |
|  |  | Pb | 295 | 0.00** | 1465 | 0.00** | 11.5 | 0.00** |
|  |  | AMF × Pb | 12.3 | 0.00** | 33.8 | 0.00** | 0.89 | 0.43ns |
|  | Neighbor | | 8.91 | 0.01** | 29.4 | 0.00** | 0.02 | 0.90ns |
|  | AMF | | 26.7 | 0.00** | 225 | 0.00** | 23.6 | 0.00** |
|  | Pb | | 485 | 0.00** | 2490 | 0.00** | 24.9 | 0.00** |
|  | Neighbor × AMF | | 0.66 | 0.42ns | 0.06 | 0.81ns | 0.94 | 0.34ns |
|  | Neighbor × Pb | | 2.46 | 0.10ns | 7.90 | 0.00** | 0.06 | 0.94ns |
|  | AMF × Pb | | 21.3 | 0.00** | 65.5 | 0.00** | 4.55 | 0.02* |
|  | Neighbor × AMF × Pb | | 0.05 | 0.95ns | 0.22 | 0.81ns | 0.87 | 0.43ns |
|  |  | |  |  |  |  |  |  |
| M | M | AMF | 8.12 | 0.01* | 29.1 | 0.00** | 12.6 | 0.00** |
|  |  | Pb | 276 | 0.00** | 604 | 0.00** | 7.77 | 0.00** |
|  |  | AMF × Pb | 12.6 | 0.00** | 7.05 | 0.01** | 0.50 | 0.61 |
|  | R+M | AMF | 8.53 | 0.01** | 64.3 | 0.00** | 8.31 | 0.01** |
|  |  | Pb | 172 | 0.00** | 1196 | 0.00** | 4.21 | 0.03* |
|  |  | AMF × Pb | 6.66 | 0.01** | 16.9 | 0.00** | 0.45 | 0.64ns |
|  | Neighbor | | 8.01 | 0.01** | 9.58 | 0.00** | 0.78 | 0.39ns |
|  | AMF | | 16.2 | 0.00** | 83.6 | 0.00** | 19.1 | 0.00** |
|  | Pb | | 408 | 0.00** | 1638 | 0.00** | 10.6 | 0.00** |
|  | Neighbor × AMF | | 0.63 | 0.43ns | 0.31 | 0.58ns | 0.17 | 0.69ns |
|  | Neighbor × Pb | | 1.98 | 0.15ns | 2.49 | 0.10ns | 0.06 | 0.94ns |
|  | AMF × Pb | | 17.0 | 0.00** | 20.8 | 0.00** | 0.80 | 0.46ns |
|  | Neighbor × AMF × Pb | | 0.16 | 0.85ns | 0.45 | 0.64ns | 0.13 | 0.88ns |
|  |  | |  |  |  |  |  |  |
| L | L | AMF | 7.94 | 0.01* | 38.1 | 0.00** | 7.02 | 0.02* |
|  |  | Pb | 111 | 0.00** | 361 | 0.00** | 2.09 | 0.15ns |
|  |  | AMF × Pb | 8.64 | 0.00** | 9.34 | 0.00** | 2.51 | 0.11ns |
|  | R+L | AMF | 12.7 | 0.00** | 43.8 | 0.00** | 9.17 | 0.01** |
|  |  | Pb | 198 | 0.00** | 576 | 0.00** | 6.42 | 0.01** |
|  |  | AMF × Pb | 13.1 | 0.00** | 10.6 | 0.00** | 1.46 | 0.26ns |
|  | Neighbor | | 5.81 | 0.02* | 11.7 | 0.00** | 0.34 | 0.57ns |
|  | AMF | | 19.9 | 0.00** | 80.9 | 0.00** | 16.1 | 0.00** |
|  | Pb | | 294 | 0.00** | 904 | 0.00** | 7.62 | 0.00** |
|  | Neighbor × AMF | | 0.03 | 0.86ns | 0.10 | 0.76ns | 0.03 | 0.87ns |
|  | Neighbor × Pb | | 1.46 | 0.25ns | 2.67 | 0.08ns | 0.66 | 0.52ns |
|  | AMF × Pb | | 21.0 | 0.00** | 19.7 | 0.00** | 3.77 | 0.03* |
|  | Neighbor × AMF × Pb | | 0.02 | 0.98ns | 0.11 | 0.90ns | 0.26 | 0.77ns |

**Table S12.** Leaf net photosynthetic rate (*P_n_*), stomatal conductance (*g_s_*), intercellular CO_2_ concentration (*C_i_*), Chlorophyll (Chl) *a* and Chl *b* contents of *R. pseudoacacia* in different treatments. Values are means ± SD of four replicates. Monocultures of *R. pseudoacacia*, *T. pretense*, *M. sativa* and *L. perenne* are represented by R, T, M and L, respectively. Co-culture planting pattern is represented by the two co-culture species connected with the plus sign (+). The results are reported as the mean (n = 4) ± SD. Different letters indicate that significant differences were detected in these parameters of each plant species grown in one planting pattern but at different Pb levels and AMF inoculation status by Duncan's multiple-range tests (*P* < 0.05).

| Planting  pattern | AMF | Pb  level | Photosynthetic parameters | | | Chlorophyll | | | |
| --- | --- | --- | --- | --- | --- | --- | --- | --- | --- |
|  |  |  | *P*_n_  μmol CO_2_ m^-1^ s^-1^ | *g*_s_  mmol H_2_O m^-1^ s^-1^ | *C*_i_  μmol CO_2_ mol^-1^ | a  (mg g^-1^) | b  (mg g^-1^) | a + b  (mg g^-1^) | a/b |
| R | -AMF | Pb0 | 8.78±1.02b | 196±20.4b | 151±21.8c | 3.31±0.11b | 1.56±0.03a | 4.90±0.13b | 2.14±0.04bc |
|  |  | Pb500 | 6.69±1.01c | 141±11.5c | 202±18.5b | 3.07±0.19c | 1.53±0.04a | 4.60±0.17c | 2.00±0.16c |
|  |  | Pb1500 | 4.91±0.66d | 96.4±14.3d | 247±16.5a | 2.60±0.15d | 1.55±0.07a | 4.15±0.21d | 1.68±0.06d |
|  | +AMF | Pb0 | 10.9±1.31a | 222±20.1a | 120±17.3d | 3.81±0.12a | 1.59±0.02a | 4.82±0.12a | 2.04±0.09a |
|  |  | Pb500 | 8.08±0.69ab | 176±22.8b | 180±13.1b | 3.55±0.19b | 1.58±0.03a | 4.53±0.17b | 1.92±0.15ab |
|  |  | Pb1500 | 7.12±0.77c | 134±13.9c | 191±22.8b | 3.08±0.23c | 1.55±0.04a | 4.35±0.22c | 1.87±0.16c |
| R+T | -AMF | Pb0 | 9.83±0.61b | 219±22.5a | 154±14.3d | 3.23±0.23bc | 1.59±0.03a | 4.96±0.21bc | 2.12±0.18bc |
|  |  | Pb500 | 8.18±0.26c | 165±20.0b | 175±18.9ab | 2.98±0.10cd | 1.55±0.03ab | 4.68±0.10cd | 1.94±0.08c |
|  |  | Pb1500 | 6.52±0.58d | 131±15.2c | 200±19.6a | 2.83±0.12d | 1.52±0.03c | 4.36±0.14d | 1.87±0.07c |
|  | +AMF | Pb0 | 11.6±1.09a | 209±17.0a | 115±22.3c | 3.66±0.22a | 1.59±0.01a | 4.02±0.21a | 1.74±0.14a |
|  |  | Pb500 | 10.1±0.68b | 176±8.75b | 142±31.6bc | 3.51±0.40ab | 1.59±0.01a | 3.87±0.38ab | 1.62±0.27ab |
|  |  | Pb1500 | 9.20±1.26bc | 154±13.7bc | 173±10.2ab | 3.23±0.24bc | 1.54±0.06ab | 3.75±0.25bc | 1.58±0.18abc |
| R+M | -AMF | Pb0 | 9.76±1.10bc | 216±29.6a | 138±10.5c | 3.37±0.12b | 1.59±0.03a | 5.40±0.13b | 2.39±0.08b |
|  |  | Pb500 | 8.68±0.66cd | 167±19.7b | 181±17.6b | 3.09±0.19cd | 1.60±0.06a | 5.12±0.19b | 2.25±0.14c |
|  |  | Pb1500 | 7.60±1.01d | 124±13.4c | 218±12.2a | 2.84±0.21d | 1.52±0.03b | 4.63±0.22c | 1.99±0.13c |
|  | +AMF | Pb0 | 11.2±1.19a | 210±21.0a | 92.1±6.07d | 3.67±0.20a | 1.59±0.03a | 5.25±0.22a | 2.31±0.12a |
|  |  | Pb500 | 10.4±0.58ab | 188±18.1ab | 135±21.6c | 3.38±0.17b | 1.54±a0.03b | 5.10±0.19b | 2.22±0.07ab |
|  |  | Pb1500 | 8.87±0.24cd | 150±11.6bc | 192±17.5b | 3.25±0.12bc | 1.51±0.04b | 4.77±0.13bc | 2.10±0.09b |
| R+L | -AMF | Pb0 | 6.69±0.42cd | 141±12.8ab | 221±16.2ab | 2.55±0.14bc | 1.47±0.01a | 5.26±0.15bc | 2.32±0.10bc |
|  |  | Pb500 | 6.99±0.69bc | 126±16.1c | 218±37.6ab | 2.39±0.14cd | 1.47±0.02a | 4.92±0.14cd | 2.19±0.10cd |
|  |  | Pb1500 | 5.85±0.50d | 124±13.9c | 234±19.0a | 2.29±0.08d | 1.45±0.04a | 4.76±0.09d | 2.14±0.06d |
|  | +AMF | Pb0 | 8.36±0.85a | 152±16.3a | 198±17.3bc | 2.96±0.17a | 1.47±0.03a | 4.43±0.19a | 2.01±0.10a |
|  |  | Pb500 | 7.83±0.69ab | 159±14.4a | 184±16.2c | 2.96±0.07a | 1.45±0.03a | 4.41±0.08a | 2.04±0.05a |
|  |  | Pb1500 | 7.06±0.21bc | 143±11.2ab | 188±5.35bc | 2.65±0.15b | 1.46±0.04a | 4.11±0.14b | 1.82±0.13b |

**Table S13.** Multiple ANOVA comparisons of leaf net photosynthetic rate (*P_n_*), stomatal conductance (*g_s_*), intercellular CO_2_ concentration (*C_i_*) and chlorophyll content of *R. pseudoacacia* affected by neighbor plant (neighbor), AMF inoculation (AMF), Pb level (Pb) and their interactions in different treatments. Monocultures of *R. pseudoacacia*, *T. pretense*, *M. sativa* and *L. perenne* are represented by R, T, M and L, respectively. Co-culture planting pattern is represented by the two co-culture species connected with the plus sign (+). (** *P* < 0.01; * *P* < 0.05; ns, no significance).

| Planting  pattern | Significance | Photosynthetic parameters | | | | | | Chlorophyll | | | | | | | |
| --- | --- | --- | --- | --- | --- | --- | --- | --- | --- | --- | --- | --- | --- | --- | --- |
|  |  | *P_n_* | | *g_s_* | | *C_i_* | | a | | b | | a+b | | a/b | |
|  |  | *F* | *P* | *F* | *P* | *F* | *P* | *F* | *P* | *F* | *P* | *F* | *P* | *F* | *P* |
| Mono | AMF | 25.0 | 0.00** | 21.3 | 0.00** | 22.8 | 0.00** | 48.1 | 0.00** | 2.20 | 0.16ns | 50.2 | 0.00** | 30.1 | 0.00** |
|  | Pb | 34.4 | 0.00** | 56.6 | 0.00** | 41.8 | 0.00** | 38.9 | 0.00** | 1.09 | 0.36ns | 39.4 | 0.00** | 26.1 | 0.00** |
|  | AMF × Pb | 0.47 | 0.63ns | 0.20 | 0.82ns | 1.84 | 0.19ns | 0.00 | 1.00ns | 0.65 | 0.53ns | 0.03 | 0.97ns | 0.18 | 0.84ns |
| R+T | AMF | 40.4 | 0.00** | 1.44 | 0.25ns | 15.4 | 0.00** | 21.8 | 0.00** | 1.82 | 0.19ns | 24.3 | 0.00** | 15.4 | 0.00** |
|  | Pb | 24.6 | 0.00** | 36.3 | 0.00** | 12.6 | 0.00** | 6.13 | 0.01** | 6.88 | 0.01ns | 8.32 | 0.00** | 2.56 | 0.11ns |
|  | AMF × Pb | 0.72 | 0.50ns | 2.07 | 0.16ns | 0.18 | 0.84ns | 0.18 | 0.84ns | 0.66 | 0.53ns | 0.26 | 0.77ns | 0.06 | 0.94ns |
| Neighbor | | 34.3 | 0.00** | 8.97 | 0.01** | 15.1 | 0.00** | 0.00 | 0.97ns | 0.00 | 0.97ns | 0.00 | 0.98ns | 0.00 | 0.95ns |
| AMF | | 63.10 | 0.00** | 17.4 | 0.00** | 37.4 | 0.00** | 61.2 | 0.00** | 4.01 | 0.05ns | 66.9 | 0.00** | 41.0 | 0.00** |
| Pb | | 58.8 | 0.00** | 92.1 | 0.00** | 48.0 | 0.00** | 31.5 | 0.00** | 5.79 | 0.01** | 36.6 | 0.00** | 18.3 | 0.00** |
| Neighbor × AMF | | 0.16 | 0.69ns | 6.33 | 0.02* | 0.08 | 0.77ns | 0.04 | 0.84ns | 0.09 | 0.77ns | 0.07 | 0.80ns | 0.00 | 0.95ns |
| Neighbor × Pb | | 1.48 | 0.24ns | 1.85 | 0.17ns | 3.45 | 0.04* | 2.65 | 0.08ns | 1.05 | 0.36ns | 2.10 | 0.14ns | 3.25 | 0.05ns |
| AMF × Pb | | 0.84 | 0.44ns | 1.71 | 0.19ns | 0.55 | 0.58ns | 0.12 | 0.89ns | 0.81 | 0.45ns | 0.26 | 0.78ns | 0.00 | 1.00ns |
| Neighbor × AMF × Pb | | 0.32 | 0.73ns | 0.46 | 0.64ns | 1.30 | 0.29ns | 0.12 | 0.89ns | 0.50 | 0.61ns | 0.10 | 0.90ns | 0.20 | 0.82ns |
|  | |  |  |  |  |  |  |  |  |  |  |  |  |  |  |
| R+M | AMF | 17.8 | 0.00** | 2.89 | 0.11ns | 40.0 | 0.00** | 22.4 | 0.00** | 1.68 | 0.21ns | 17.0 | 0.00** | 30.0 | 0.00** |
|  | Pb | 13.6 | 0.00** | 29.7 | 0.00** | 70.4 | 0.00** | 15.6 | 0.00** | 7.61 | 0.00** | 17.4 | 0.00** | 8.34 | 0.00** |
|  | AMF × Pb | 0.15 | 0.86ns | 1.54 | 0.24ns | 1.18 | 0.33ns | 0.26 | 0.77ns | 1.46 | 0.26ns | 0.42 | 0.66ns | 0.28 | 0.76ns |
| Neighbor | | 41.3 | 0.00** | 7.64 | 0.01** | 20.6 | 0.00** | 0.26 | 0.61ns | 0.17 | 0.69ns | 0.16 | 0.70ns | 0.49 | 0.49ns |
| AMF | | 42.7 | 0.00** | 18.8 | 0.00** | 59.2 | 0.00** | 67.7 | 0.00** | 0.05 | 0.83ns | 61.5 | 0.00** | 59.8 | 0.00** |
| Pb | | 45.6 | 0.00** | 82.3 | 0.00** | 105 | 0.00** | 50.6 | 0.00** | 6.55 | 0.00** | 53.2 | 0.00** | 31.4 | 0.00** |
| Neighbor × AMF | | 0.68 | 0.42ns | 3.25 | 0.08ns | 0.09 | 0.77ns | 2.13 | 0.15ns | 3.88 | 0.06ns | 3.28 | 0.08ns | 0.22 | 0.64ns |
| Neighbor × Pb | | 4.06 | 0.03* | 1.05 | 0.36ns | 1.24 | 0.30ns | 3.28 | 0.05* | 1.59 | 0.22ns | 2.08 | 0.14ns | 5.20 | 0.01** |
| AMF × Pb | | 0.07 | 0.94ns | 1.50 | 0.24ns | 0.18 | 0.83ns | 0.15 | 0.87ns | 0.28 | 0.76ns | 0.13 | 0.88ns | 0.32 | 0.73ns |
| Neighbor × AMF × Pb | | 0.58 | 0.56ns | 0.39 | 0.68ns | 2.96 | 0.06ns | 0.12 | 0.89ns | 1.77 | 0.19ns | 0.36 | 0.70ns | 0.12 | 0.89ns |
|  | |  |  |  |  |  |  |  |  |  |  |  |  |  |  |
| R+L | AMF | 31.1 | 0.00** | 12.3 | 0.00** | 16.0 | 0.00** | 68.3 | 0.00** | 0.22 | 0.65ns | 61.3 | 0.00** | 66.9 | 0.00** |
|  | Pb | 3.31 | 0.06ns | 1.68 | 0.21ns | 0.56 | 0.58ns | 9.66 | 0.00** | 0.44 | 0.65ns | 9.71 | 0.00** | 7.55 | 0.00** |
|  | AMF × Pb | 1.42 | 0.27ns | 1.27 | 0.30ns | 0.59 | 0.57ns | 1.30 | 0.30ns | 0.43 | 0.66ns | 0.92 | 0.42ns | 1.81 | 0.19ns |
| Neighbor | | 7.45 | 0.01** | 18.5 | 0.00** | 20.0 | 0.00** | 193 | 0.00** | 94.1 | 0.00** | 244 | 0.00** | 76.3 | 0.00** |
| AMF | | 48.3 | 0.00** | 33.5 | 0.00** | 37.9 | 0.00** | 111 | 0.00** | 0.89 | 0.35ns | 108 | 0.00** | 87.1 | 0.00** |
| Pb | | 39.0 | 0.00** | 44.2 | 0.00** | 18.4 | 0.00** | 46.5 | 0.00** | 1.50 | 0.24ns | 46.9 | 0.00** | 32.8 | 0.00** |
| Neighbor × AMF | | 2.23 | 0.14ns | 1.92 | 0.17ns | 0.04 | 0.85ns | 0.14 | 0.72ns | 2.20 | 0.15ns | 0.48 | 0.49ns | 0.41 | 0.52ns |
| Neighbor × Pb | | 14.4 | 0.00** | 25.8 | 0.00** | 19.1 | 0.00** | 9.05 | 0.00** | 0.25 | 0.78ns | 9.01 | 0.00** | 5.68 | 0.01** |
| AMF × Pb | | 1.10 | 0.34ns | 0.97 | 0.39ns | 1.86 | 0.17ns | 0.49 | 0.62ns | 0.17 | 0.84ns | 0.53 | 0.60ns | 0.35 | 0.71ns |
| Neighbor × AMF × Pb | | 0.13 | 0.88ns | 0.28 | 0.76ns | 0.42 | 0.66ns | 0.50 | 0.61ns | 0.98 | 0.38ns | 0.22 | 0.81ns | 1.21 | 0.31ns |

**Table S14.** Correlation coefficients among plant height, total dry weight, macronutrient concentrations and photosynthetic parameters of *R. pseudoacacia* in different treatments. Monocultures of *R. pseudoacacia*, *T. pretense*, *M. sativa* and *L. perenne* are represented by R, T, M and L, respectively. Co-culture planting pattern is represented by the two co-culture species connected with the plus sign (+). (** *P* < 0.01; * *P* < 0.05; ns, no significance).

| Parameters | | Biomass | | | | | | | |
| --- | --- | --- | --- | --- | --- | --- | --- | --- | --- |
|  |  | -AMF | | | | +AMF | | | |
|  |  | R | R+T | R+M | R+L | R | R+T | R+M | R+L |
| Macronutrient | |  |  |  |  |  |  |  |  |
| N | Shoot | 0.83** | 0.78** | 0.75** | 0.78** | 0.79** | 0.61* | 0.57ns | 0.64* |
|  | Root | 0.80** | 0.70* | 0.72** | 0.62* | 0.79** | 0.64* | 0.31ns | 0.38** |
| P | Shoot | 0.93** | 0.87** | 0.81** | 0.78** | 0.87** | 0.63* | 0.54ns | 0.64* |
|  | Root | 0.92** | 0.84** | 0.80** | 0.89** | 0.85** | 0.61* | 0.50ns | 0.44ns |
| S | Shoot | 0.94** | 0.76** | 0.89** | 0.55ns | 0.72** | 0.79** | 0.84* | 0.19ns |
|  | Root | 0.92** | 0.54ns | 0.72** | 0.32ns | 0.82** | 0.68* | 0.66* | 0.02ns |
| Na | Shoot | 0.09ns | -0.18ns | -0.09ns | -0.04ns | 0.17ns | 0.09ns | -0.13ns | -0.05ns |
|  | Root | 0.08ns | -0.07ns | -0.04ns | 0.08ns | 0.47ns | 0.26ns | -0.18ns | -0.17ns |
| K | Shoot | -0.12ns | -0.19ns | 0.00ns | 0.14ns | 0.11ns | 0.07ns | 0.28ns | -0.08ns |
|  | Root | 0.15ns | -0.10ns | 0.22ns | -0.11ns | 0.17ns | 0.00ns | -0.15ns | -0.02ns |
| Ca | Shoot | -0.49ns | -0.03ns | -0.33ns | -0.16ns | -0.20ns | 0.10ns | 0.41ns | -0.05ns |
|  | Root | 0.02ns | 0.13ns | -0.05ns | 0.16ns | -0.06ns | 0.10ns | 0.00ns | 0.06ns |
| Mg | Shoot | 0.78** | 0.52ns | 0.32ns | 0.65* | 0.49ns | 0.66* | 0.36ns | -0.05ns |
|  | Root | 0.81** | 0.61* | 0.74* | 0.56ns | 0.69* | 0.29ns | -0.09ns | 0.32ns |
| N/P | Shoot | -0.63* | -0.48ns | -0.33ns | -0.10ns | -0.56ns | -0.40ns | -0.14ns | 0.07ns |
|  | Root | 0.33ns | -0.61* | -0.28ns | -0.41ns | -0.46ns | -0.32ns | -0.51 | -0.01ns |
| N_shoot_: N_root_ | | 0.22ns | 0.42ns | 0.17ns | 0.15ns | 0.24ns | -0.10ns | 0.59* | 0.14ns |
| P_shoot_: P_root_ | | 0.66* | 0.34ns | 0.38ns | -0.28ns | 0.27ns | -0.22ns | 0.33ns | 0.03ns |
| Photosynthesis | |  |  |  |  |  |  |  |  |
| *P_n_* | | 0.89** | 0.76** | 0.52ns | 0.38ns | 0.63* | 0.45ns | 0.43ns | 0.63* |
| *g_s_* | | 0.88** | 0.69* | 0.70* | 0.50ns | 0.85ns | 0.51ns | 0.51ns | 0.08ns |
| *C_i_* | | -0.89** | -0.60* | -0.68* | -0.14ns | -0.75* | -0.52ns | -0.49ns | 0.43ns |
| Chlorophyll | |  |  |  |  |  |  |  |  |
| a | | 0.91** | 0.55ns | 0.58* | 0.62* | 0.87* | 0.42ns | 0.17ns | 0.35ns |
| b | | 0.04ns | 0.57ns | 0.72** | 0.23ns | 0.42ns | 0.57ns | 0.30ns | 0.26ns |
| a+b | | 0.89** | 0.60* | 0.64* | 0.63* | 0.88** | 0.47ns | 0.20ns | 0.38ns |
| a/b | | 0.91** | 0.38ns | 0.37ns | 0.57ns | 0.84** | 0.28ns | 0.07ns | 0.27ns |

**Table S15.** Correlation coefficients among total dry weight and macronutrient concentrations in shoots and roots of *T. pretense*, *M. sativa* and *L. perenne* in different treatments. Monocultures of *R. pseudoacacia*, *T. pretense*, *M. sativa* and *L. perenne* are represented by R, T, M and L, respectively. Co-culture planting pattern is represented by the two co-culture species connected with the plus sign (+). (** *P* < 0.01; * *P* < 0.05; ns, no significance).

| Macronutrient | | -AMF | | | | | | +AMF | | | | | |
| --- | --- | --- | --- | --- | --- | --- | --- | --- | --- | --- | --- | --- | --- |
|  |  | T | | M | | L | | T | | M | | L | |
|  |  | T | R+T | M | R+M | L | R+L | T | R+T | M | R+M | L | R+L |
| N | Shoot | 0.77** | 0.77** | 0.46ns | 0.58* | -0.22ns | 0.43ns | 0.58* | 0.66* | 0.66* | 0.38ns | 0.52ns | -0.36ns |
|  | Root | 0.26ns | 0.50ns | 0.37ns | 0.21ns | 0.23ns | 0.53ns | 0.23ns | 0.24ns | 0.49ns | 0.21ns | 0.39ns | 0.19ns |
| P | Shoot | 0.72** | 0.69** | 0.66ns | 0.54ns | -0.60* | -0.76** | 0.68* | 0.42ns | 0.20ns | 0.46ns | 0.16ns | 0.39ns |
|  | Root | 0.76** | 0.52ns | 0.55ns | 0.55ns | -0.34ns | 0.80** | 0.62* | 0.33ns | 0.33ns | 0.41ns | -0.14ns | 0.20ns |
| S | Shoot | 0.58* | 0.58* | 0.36ns | 0.34ns | 0.10ns | -0.10ns | 0.29ns | 0.56ns | 0.40ns | 0.51ns | -0.37ns | -0.32ns |
|  | Root | 0.29ns | 0.25ns | 0.46ns | 0.28ns | -0.44ns | 0.18ns | -0.02ns | 0.21ns | 0.54ns | 0.70* | -0.34ns | -0.16ns |
| Mg | Shoot | 0.67* | 0.73** | 0.55ns | 0.42ns | -0.17ns | -0.64* | 0.33ns | 0.48ns | 0.35ns | -0.20ns | -0.03ns | 0.39ns |
|  | Root | 0.65* | 0.25ns | 0.40ns | 0.09ns | -0.25ns | -0.23ns | 0.20ns | 0.18ns | 0.02ns | -0.27ns | -0.27ns | 0.01ns |
| N/P | Shoot | -0.21ns | 0.07ns | -0.31ns | -0.11ns | -0.02ns | 0.66* | 0.15ns | 0.50ns | 0.59* | 0.11ns | 0.43ns | -0.45ns |
|  | Root | -0.81** | -0.04ns | -0.38ns | -0.39ns | 0.41ns | 0.47ns | -0.20ns | 0.14ns | 0.16ns | -0.04ns | 0.39ns | 0.08ns |
| N_shoot_: N_root_ | | 0.34ns | 0.29ns | 0.23ns | 0.34ns | -0.59* | 0.74** | 0.32ns | 0.45ns | 0.34ns | 0.18ns | 0.12ns | -0.45ns |
| P_shoot_: P_root_ | | -0.32ns | 0.19ns | 0.12ns | -0.07ns | -0.11ns | -0.38ns | 0.04ns | 0.14ns | -0.16ns | 0.15ns | 0.24ns | 0.18ns |


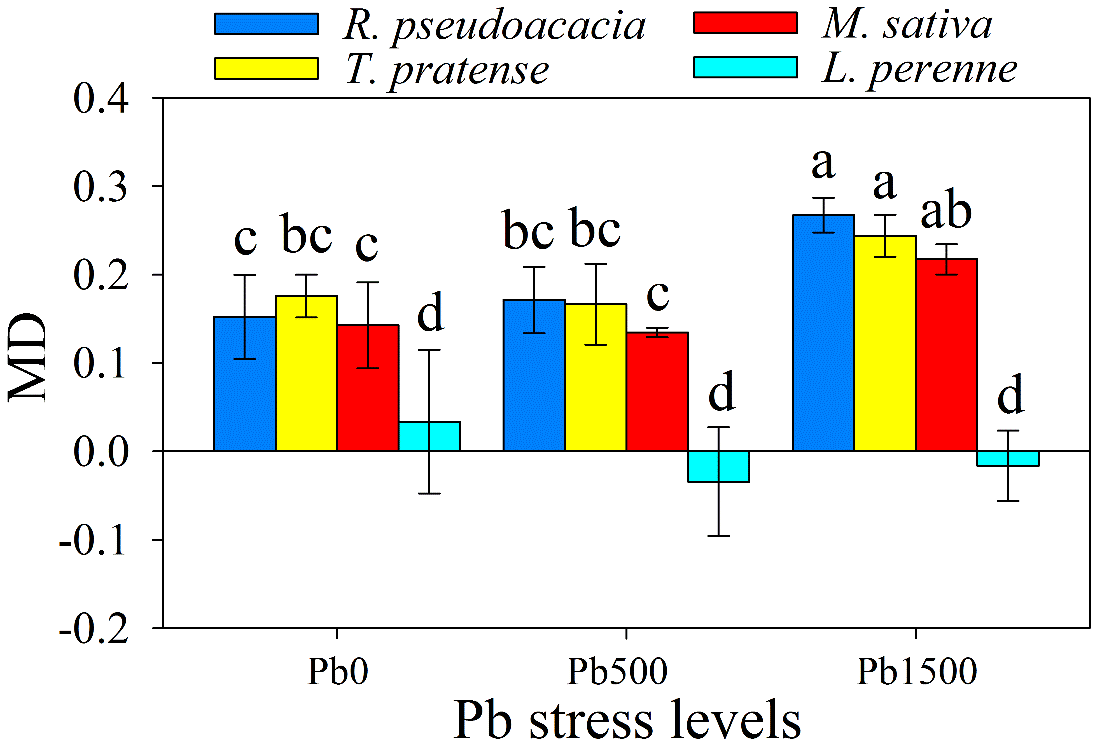


**Figure S1.** Mycorrhizal dependency (MD) of *R. pseudoacacia*, *T. pretense*, *M. sativa* and *L. perenne* (means ± SD, n=4) in different treatments. Pb0, Pb500 and Pb1500 represent Pb concentration of 0, 500 and 1500 mg kg^-1^, respectively. Monocultures of *R. pseudoacacia*, *T. pretense*, *M. sativa* and *L. perenne* are represented by R, T, M and L, respectively. Co-culture planting pattern is represented by the two co-culture species connected with the plus sign (+). The results are reported as the mean (n = 4) ± SD. Different letters indicate that significant differences were detected in MD of each plant species at different Pb levels by Duncan's multiple-range tests (*P* < 0.05).


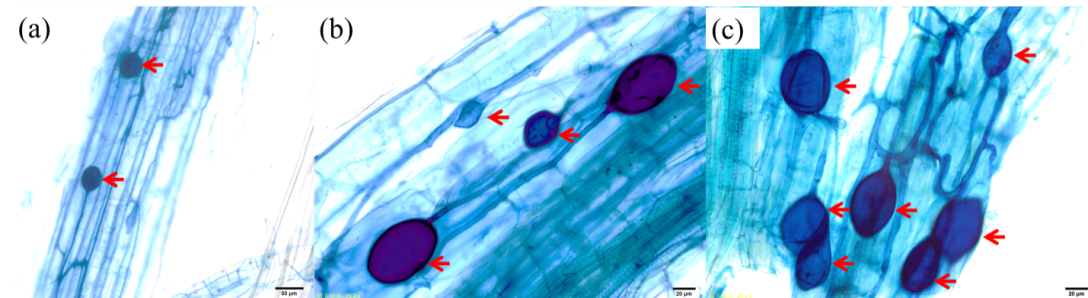


**Figure S2**. Typical AMF vesicle structures in roots of *R. pseudoacacia* which grown individually at (a) Pb0 level, (b) Pb500 level and (c) Pb1500 level. Red arrows present fungal vesicle structures. Bar presents the length of 50 μm for image (a), and 20 μm for images (b) and (c).


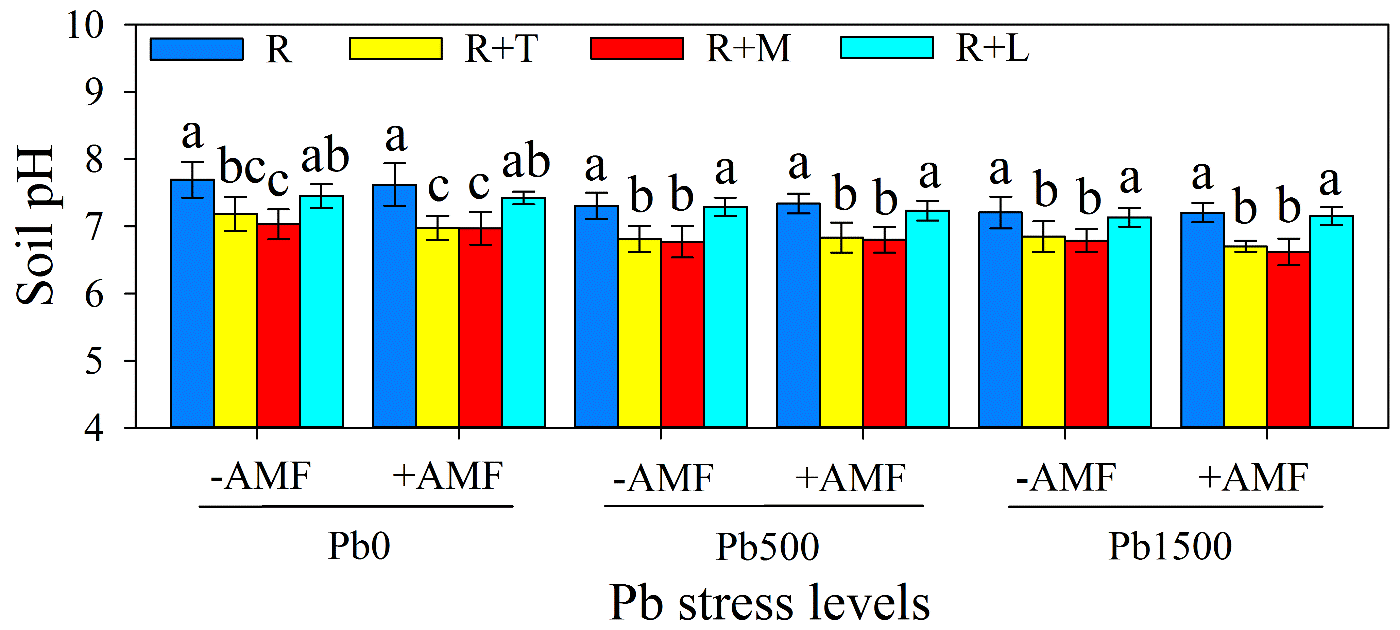


**Figure S3.** Soil pH values in rhizosphere soil of plants (means ± SD, n=4) in different planting patterns. Pb0, Pb500 and Pb1500 represent Pb concentration of 0, 500 and 1500 mg kg^-1^, respectively. Monocultures of *R. pseudoacacia*, *T. pretense*, *M. sativa* and *L. perenne* are represented by R, T, M and L, respectively. Co-culture planting pattern is represented by the two co-culture species connected with the plus sign (+). The results are reported as the mean (n = 4) ± SD. Different letters indicate that significant differences were detected in soil pH of plants grown at the same Pb levels but in different planting patterns and AMF inoculation status by Duncan's multiple-range tests (*P* < 0.05).


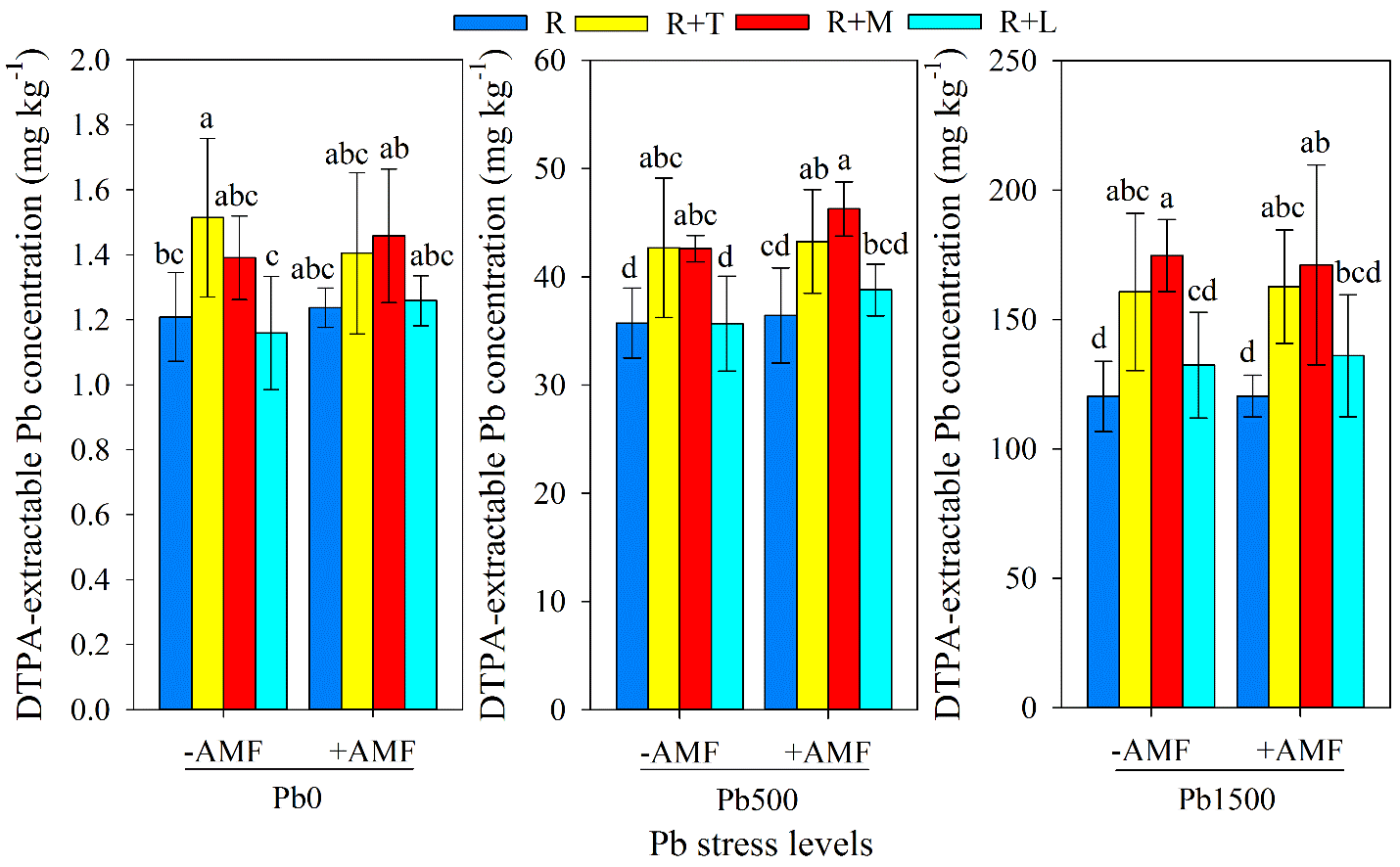


**Figure S4.** DTPA-extractable Pb concentrations in rhizosphere soil of plants (means ± SD, n=4) in different planting patterns. Pb0, Pb500 and Pb1500 represent Pb concentration of 0, 500 and 1500 mg kg^-1^, respectively. Monocultures of *R. pseudoacacia*, *T. pretense*, *M. sativa* and *L. perenne* are represented by R, T, M and L, respectively. Co-culture planting pattern is represented by the two co-culture species connected with the plus sign (+). The results are reported as the mean (n = 4) ± SD. Different letters indicate that significant differences were detected in DTPA-extractable Pb concentrations of plants grown at the same Pb levels but in different planting patterns and AMF inoculation status by Duncan's multiple-range tests (*P* < 0.05).
